# IFI27 Is a Useful Genetic Marker for Diagnosis of Immunoglobulin A Nephropathy and Membranous Nephropathy Using Peripheral Blood

**DOI:** 10.1371/journal.pone.0153252

**Published:** 2016-04-21

**Authors:** Yasuyuki Nagasawa, Daisuke Okuzaki, Eri Muso, Ryohei Yamamoto, Maki Shinzawa, Yukako Iwasaki, Hirotsugu Iwatani, Takeshi Nakanishi, Yoshitaka Isaka, Hiroshi Nojima

**Affiliations:** 1 Department of Geriatric Medicine and Nephrology, Osaka University Graduate School of Medicine, 2-2 Yamadaoka, Suita, Osaka, Japan; 2 Department of Internal Medicine, Division of Kidney and Dialysis, Hyogo College of Medicine, 1-1 Mukogawa-Cho, Nishinomiya, Hyogo, Japan; 3 DNA-chip Development Center for Infectious Diseases, Research Institute for Microbial Diseases, Osaka University, 3-1 Yamadaoka, Suita, Osaka, Japan; 4 Department of Molecular Genetics, Research Institute for Microbial Diseases, Osaka University, 3-1 Yamadaoka, Suita, Osaka, Japan; 5 Division of Nephrology and Hemodialysis, Kitano Hospital, The Tazuke Kofukai Medical Research Institute, 2-4-20 Ohgimachi, Kita-ku, Osaka, Japan; University of Florida, UNITED STATES

## Abstract

Diagnosis of chronic glomerulonephritis (CGN) depends primarily on renal biopsy, which is expensive and requires hospitalization, creating a demand for noninvasive diagnostic method for this disease. We used DNA microarray analysis to search for genes whose expression levels in peripheral blood mononuclear cells (PBMCs) could distinguish between patients with CGN and healthy volunteers (HVs). We selected immunoglobulin A nephropathy (IgAN) and membranous nephropathy (MN) as typical forms of CGN. The mRNA level of the gene encoding interferon (IFN)-alpha-inducible protein 27, *IFI27*, which is preferentially expressed in podocytes of glomeruli, was lower in PBMCs of IgAN and MN patients than in those of HVs. This result was confirmed by quantitative real-time reverse transcription polymerase chain reaction (qRT-PCR). Moreover, qRT-PCR analysis revealed that the *IFI27* mRNA level was reduced in PBMCs of patients with other types of chronic glomerulonephritis. IFI27 immunohistochemical staining of biopsied specimens also confirmed reduced expression of IFI27 protein in IgAN and MN patients. Based on these results, we propose that IFI27 could serve as a noninvasive diagnostic marker for CGNs using peripheral blood.

## Introduction

Immunoglobulin A nephropathy (IgAN), one of the most prevalent forms of chronic glomerulonephritis (CGN) among Asians [1] and Caucasians [2], is characterized by the presence of prominent IgA1 deposits in the glomerular mesangium [3]. The 30-year renal survival rate of IgAN is 50.3% [4], i.e., the condition has a poor renal prognosis. Several candidate factors related to the pathogenesis of IgAN have been identified, including genetic [5], [6], immune [7–9], and infectious factors [10], [11]. When IgAN patients acquire upper respiratory infections such as tonsillitis, they often manifest deteriorated urinary findings such as macroscopic hematuria, suggestive of some type of communication through the blood between the tonsillar immune system and kidney. Indeed, the number of CD16+CD56+ cells in peripheral blood increases just after tonsillectomy in IgAN patients, and these cells cause hematuria in rats. MicroRNA in peripheral blood mononuclear cells (PBMCs) of IgAN patients contributed to expression of pathogenic polymeric IgA1 [12]. Therefore, peripheral blood might be useful for diagnosis of IgAN.

On the other hand, other primary glomerular nephropathies have features that differ from those of IgAN and membranous nephropathy (MN), for example, is characterized by the formation of subepithelial immune deposits, causing abnormalities in glomerular basement membrane (GBM); the majority of cases of primary MN are associated with auto-antibodies against the podocyte antigen M-type phospholipase A2 receptor [13], [14]. Anti-neutrophil cytoplasmic antibody (ANCA)-associated glomerulonephritis (AAG) is characterized by anti-neutrophil antibody along with systemic vasculitis [15], whereas minimal change nephrotic syndrome (MCNS) is induced by an allergic mechanism [16]. Notably, the features of these glomerular diseases are reflected by certain conditions of the peripheral blood, making it possible to distinguish IgAN from other glomerular diseases by analyzing peripheral blood samples. The primary means for diagnosing IgAN is renal biopsy, which is costly and requires hospitalization; these obstacles contribute to delays in diagnosis, which may in turn be partially responsible for the poor prognosis of IgAN. Therefore, reliable noninvasive biomarkers that might be applicable in routine clinical practice are urgently required [17].

DNA microarray analysis is useful for comprehensively identifying genes that are up- or down-regulated in PBMCs; indeed, our group has successfully identified disease-specific genes in PBMCs of patients suffering from Churg-Strauss syndrome [18], Takayasu arteritis [19], and microscopic polyangiitis (MPA) [20]. We also identified several genes that are up-regulated in the tonsils of IgAN patients [7], [8]. PBMCs are useful for the diagnosis of other diseases. For example, elevated mRNA expression by human endogenous retrovirus subgroup k in PBMCs may serve as a non-invasive early disease detection marker for prostate cancer [21], and PBMC (T-cell) based assays for tuberculous meningitis in HIV-uninfected patients is useful for the early diagnosis of this disease [22]. By contrast, no such useful diagnostic marker for CGN has been reported heretofore.

In this study, we used DNA microarray analysis to search PBMC mRNA for genes whose expression levels distinguish between patients with CGN and healthy volunteers (HVs). We selected IgAN and MN as typical CGNs, and performed DNA microarray analysis on PBMC mRNA from IgAN, MN, and HVs. The mRNA level of the gene encoding interferon (IFN)-alpha-inducible protein 27 (*IFI27*) was down-regulated in PBMCs of IgAN and MN patients relative to those of HVs. IFI27 is up-regulated in PBMCs of systemic lupus erythematosus patients [23] and in inflammatory psoriatic skin and in some epithelial cancers [24]. After confirming this result by quantitative real-time reverse transcription polymerase chain reaction (qRT-PCR), we extended the qRT-PCR analysis to other kidney diseases and found that *IFI27* mRNA levels were reduced in PBMCs of many patients. We also performed immunohistochemistry (IHC) on biopsied clinical samples of IgAN and MN using an anti-IFI27 antibody and observed reduced immunostaining of IFI27 in podocytes. These results suggest that IFI27 could serve as a noninvasive biomarker in peripheral blood samples, a method that would be readily applicable in routine clinical practice.

## Results

### Identification of up- or down-regulated genes in PBMCs of IgAN and MN patients

**Fig 1:**
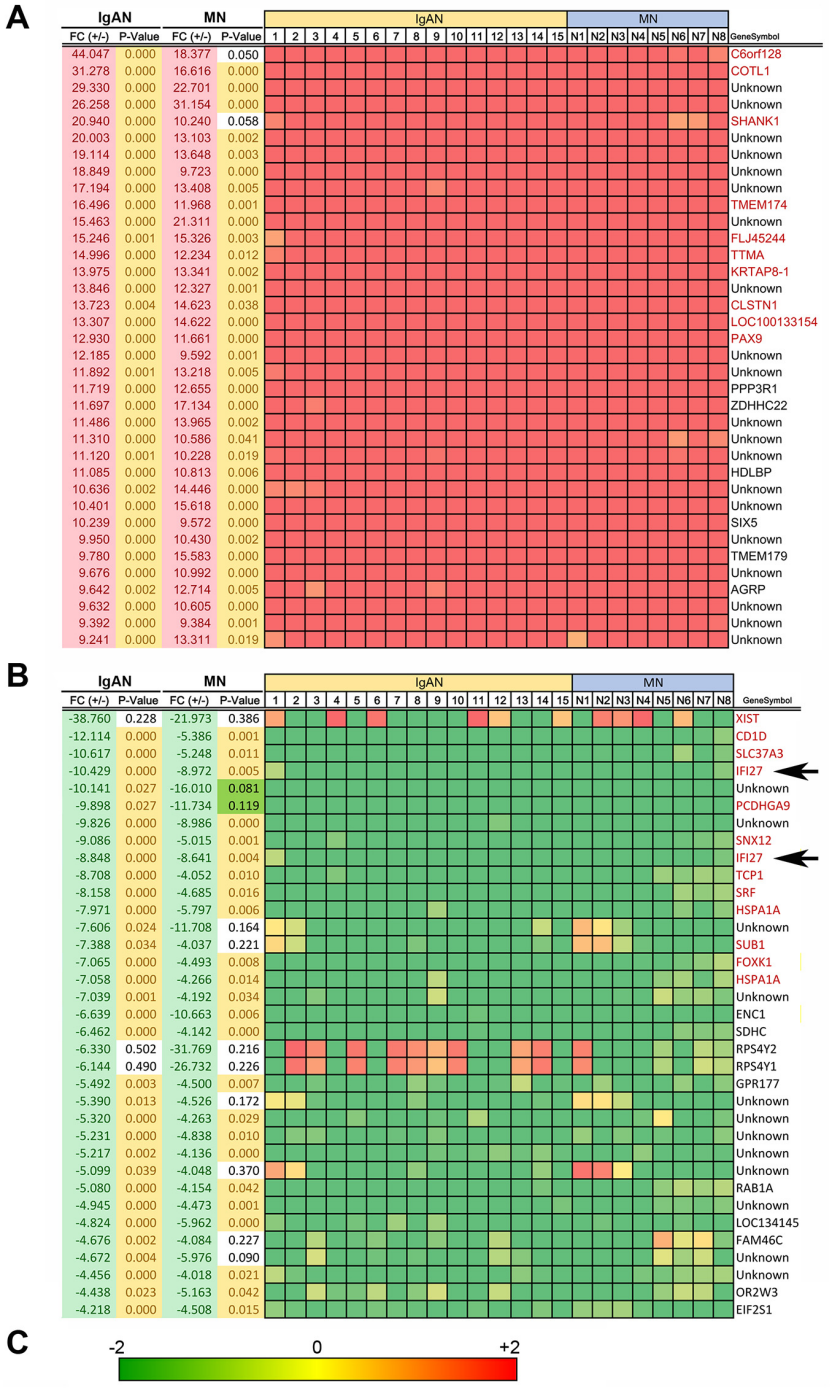
Expression profiles of genes whose mRNA levels were up- or down-regulated in the PBMCs of 15 IgAN (IgAN1- IgAN15) and eight MN (MN1-MN8) patients relative to those of HVs. **(A)** List of the top 50 genes up-regulated in most IgAN and MN patients, shown in decreasing order of fold-change values. **(B)** List of bottom 50 genes down-regulated in most IgAN and MN patients, shown in increasing order of fold-change values. “Unknown” indicates uncharacterized genes. Mosaic tile representation of each gene is also shown, with intensity gradients indicating the mean value of the expression level (log_2_ ratio): down-regulation (green) and up-regulation (red) are expressed relative to the average value in healthy volunteers (yellow). **(C)** Bar represents the standard intensity gradient. Names of genes also appearing in Fig 2 are shown in pink font. Arrows indicate *IFI27*.

**Fig 2:**
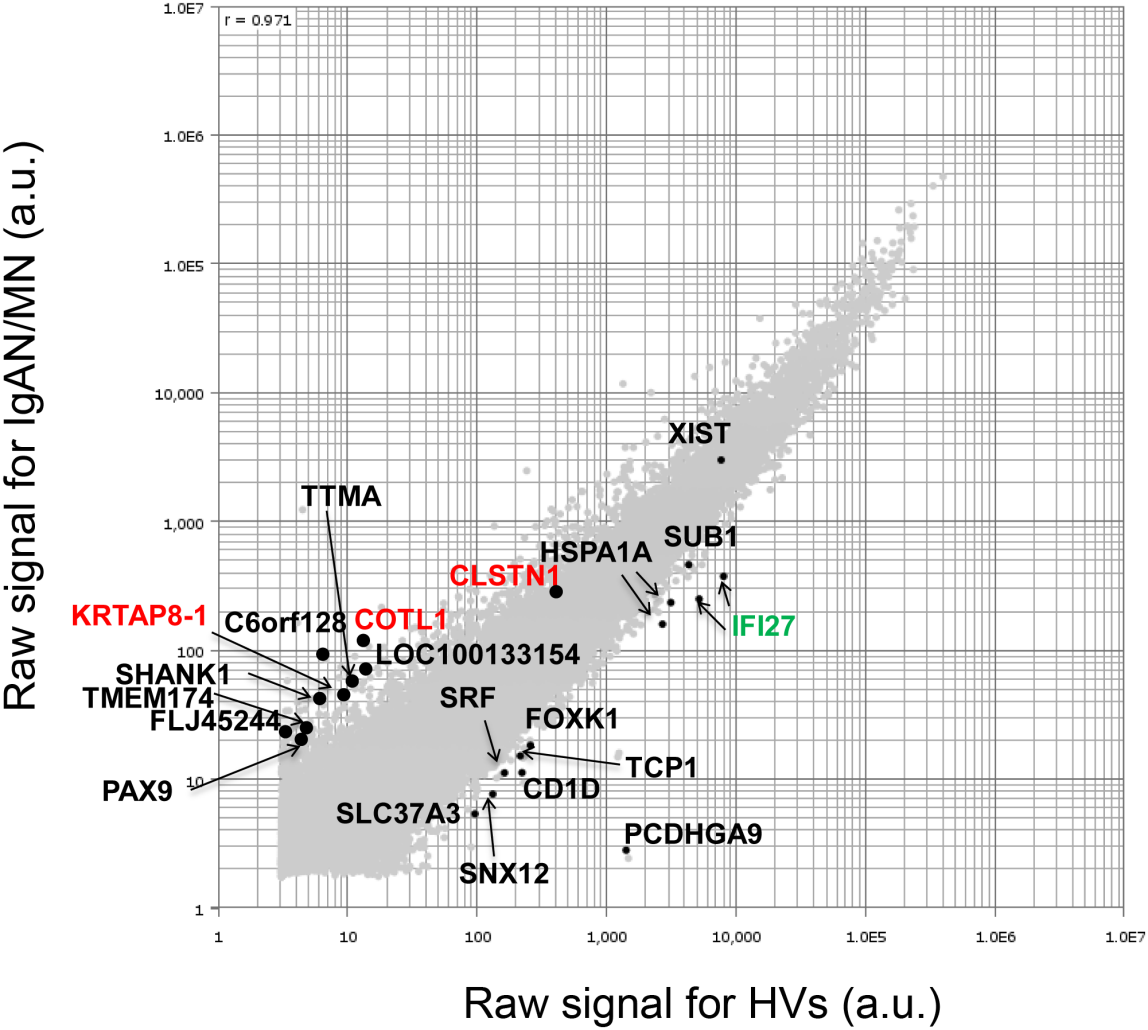
Microarray expression profiling. Scatter plot for genes highlighted in red front in Fig 1. DNA microarray data, expressed as log of signal intensity of IgAN/MN vs HVs, are plotted along the vertical and horizontal axes, respectively. Horizontal or vertical axis indicates the raw signal intensity of IgAN/MN or HVs (arbitrary units: a.u.), respectively. *CLSTN1*, *COTL*, *KRTAP8-1*, and *IFI27*, which were subjected to qRT-PCR analysis, are highlighted in red or green font.

To search for genes with elevated or reduced mRNA levels in PBMCs of CGN patients, we performed a genome-wide cDNA microarray analysis (Agilent Hu44K arrays) using RNA samples isolated from PBMCs of 15 IgAN and eight MN patients (S1 Fig). The relative intensities of the signals for each patient were compared with those of mixed HV samples [25]. To identify, at a glance, genes that were up- or down-regulated in PBMCs of IgAN patients, MN patients, or both, we ranked the up-regulated genes (fold-change > 9.0) in order of decreasing fold-change in IgAN and MN patients (Fig 1A). The top 20 up-regulated genes did not include clinically noteworthy genes (see Discussion); we disregarded *C6orf128*, *COTL1*, *SHANK1*, *TMEM174*, *TTMA*, *KRTAP8-1*, *CLSTN1*, and *PAX9* because the scatter plot revealed that their mRNA levels were too low to be physiologically significant in IgAN and/or MN patients (Fig 2).

Microarray signals sometimes yield false signals, in particular when the level of an mRNA is very low. Therefore, to perform physiologically significant comparisons, it is necessary to validate microarray by other methods such as qRT-PCR [26]. Indeed, the mRNA levels of *CLSTN*, *COTL1*, and *KRTAP8-1* assessed by qRT-PCR differed significantly among the patients (S2A–S2F Fig); moreover, the putative functions of these genes in the kidney are unknown.

We next searched for the genes that were up- or down-regulated in IgAN but not in MN patients by selecting genes with fold-change >3.0 in IgAN and <1.0 in MN (S3A Fig), or <1.0 in IgAN and >3.0 in MN (S3B Fig). However, this analysis detected no noteworthy genes. Indeed, qRT-PCR of *KRTAP5-8* yielded mRNA levels that differed from those obtained by the microarray analysis (S2G and S2H Fig).

By contrast, when we ranked genes that were down-regulated (fold-change <-5.0) in both IgAN and MN patients in order of increasing fold-change, we identified several genes that were down-regulated in all tested patients (Fig 1B). In particular, we noted *IFI27*, also referred to as interferon-stimulated gene 12a (*ISG12A*), which is expressed abundantly in podocytes of glomeruli as determined by *in situ* hybridization (ISH) and qRT-PCR [23]. We disregarded other genes with a higher rank because their mRNA levels were too low for a likely physiological role, as judged by scatter plot (Fig 2).

### *IFI27* mRNA level is reduced in PBMCs of IgAN and MN patients

**Fig 3:**
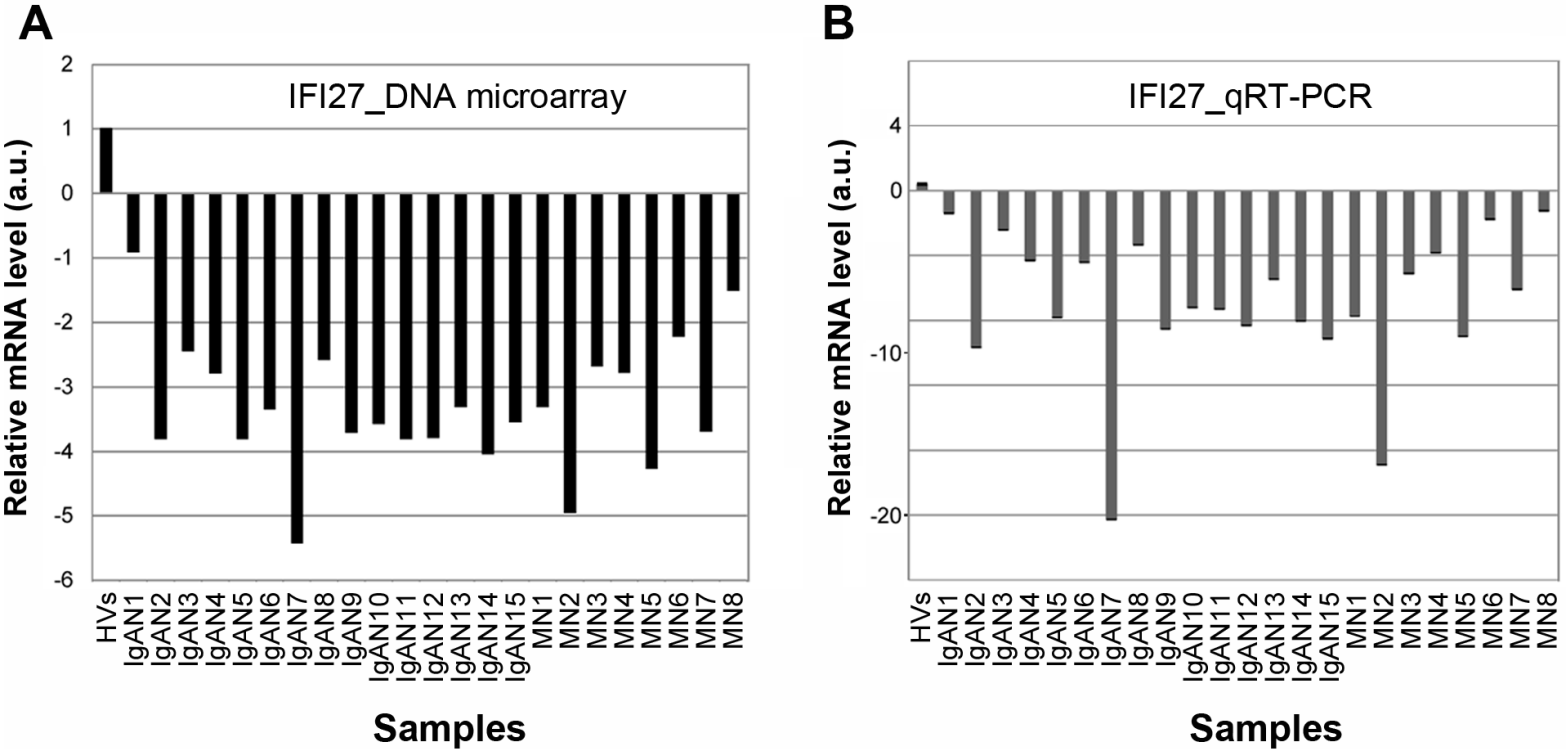
Comparison of DNA microarray and qRT-PCR data. **(A, B)** Relative *IFI27* mRNA levels for individual IgAN and MN patients determined by DNA microarray (A) or qRT-PCR (B) are shown by box graphs. Vertical axis indicates the mRNA level (arbitrary units: a.u.) relative to the value in HVs, which was fixed at 1.0 a.u.

**Fig 4:**
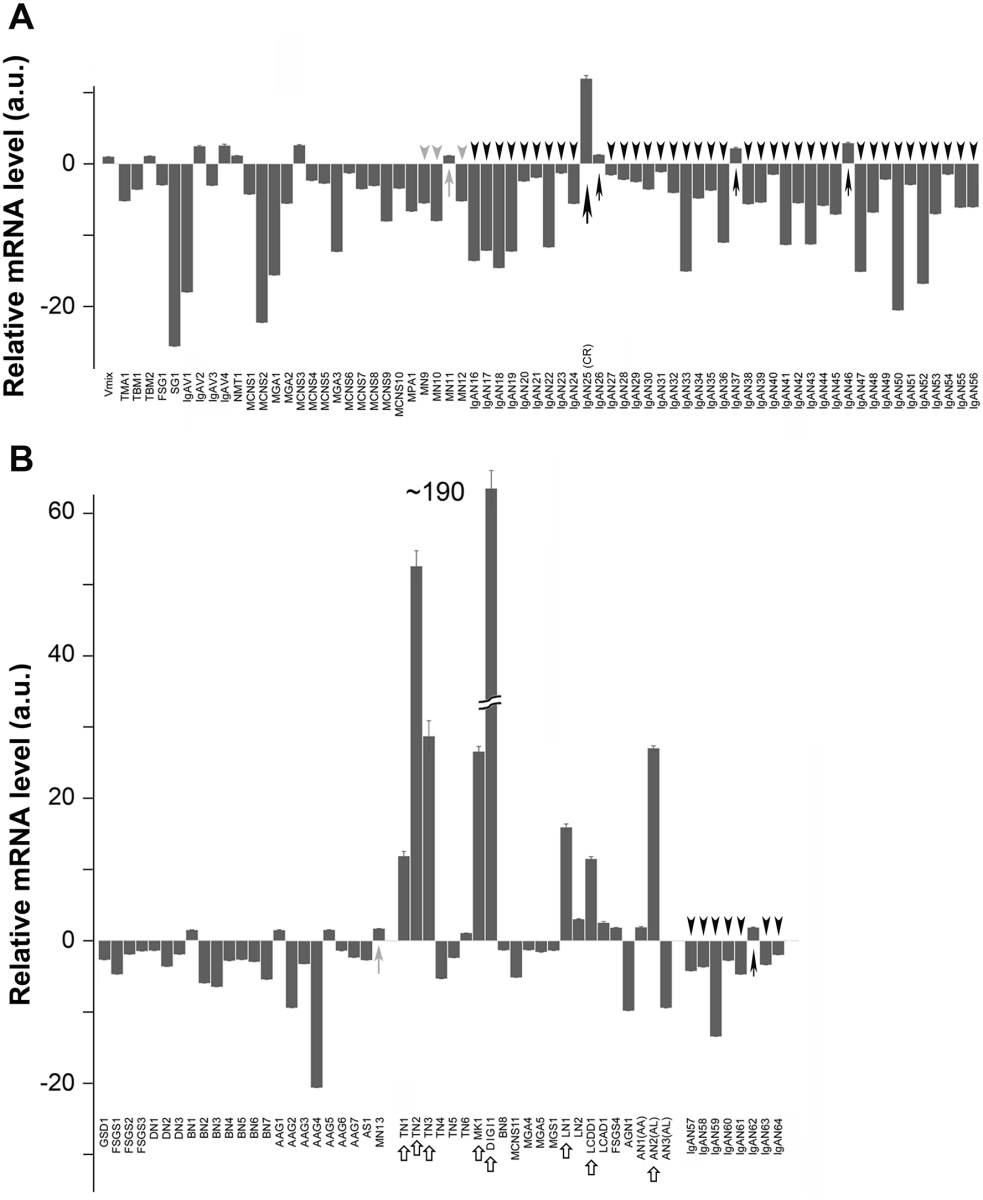
Relative *IFI27* mRNA levels of individual patients suffering from the indicated kidney diseases. **(A, B)** Box graphs show relative *IFI27* mRNA levels, determined by qRT-PCR, for the indicated patients. Horizontal axis indicates the mRNA level (arbitrary units: a.u.) relative to the value in HVs, which was fixed at 1.0 a.u. Black and gray arrowheads indicate IgAN and MN patients whose *IFI27* mRNA levels were lower than those of HVs (Vmix). Black and gray arrows indicate IgAN and MN patients whose *IFI27* mRNA levels were slightly elevated. A large black arrow indicates an IgAN25 patient who had entered complete remission at the time of blood sample collection and who had a high *IFI27* mRNA level. White arrows indicate other patients who had high *IFI27* mRNA levels.

Both DNA microarray and qRT-PCR analyses yielded similar reductions in the *IFI27* mRNA level (Fig 3), i.e., the microarray results were reproducible and validated. To determine whether the *IFI27* mRNA level is also reduced in other kidney diseases, we performed qRT-PCR analysis on mRNA obtained from PBMCs of patients suffering IgAN, MN, or other kidney diseases (Fig 4) such as thrombotic microangiopathy (TMA), thin basement membrane disease (TBM), focal segmental glomerulosclerosis (FSGS), sclerosing glomerulonephritis (SG), IgA vasculitis (IgAV), nephritis after bone marrow transplantation (NMT), minimal change nephrotic syndrome (MCNS), minor glomerular abnormalities (MGA), MPA, glycogen storage disease (GSD), diabetic nephropathy (DN), benign nephrosclerosis (BN), anti-neutrophil cytoplasmic antibody (ANCA)-associated glomerulonephritis (AAG), Alport syndrome (AS), tubulointersitial nephritis (TN), myeloma kidney (MK), drug-induced glomerular injury (DIGI), lupus nephritis (LN), light chain deposition disease (LCDD), lecithin: cholesterol acyltransferase (LCAT) deficiency (LCAD), anti-GBM glomerulonephritis (AGN), and amyloid nephropathy (AN).

The *IFI27* mRNA level was reduced in most newly examined 48 IgAN patients (black arrowheads in Fig 4), although four IgAN patients had slightly elevated levels (small black arrows). Patient IgAN25 had an exceptionally elevated *IFI27* mRNA level; medical records revealed that this patient had entered complete remission (post-tonsillectomy and steroid pulse therapy) at the time of blood collection, suggesting that *IFI27* mRNA levels (large black arrow) are tightly linked to patients’ current symptoms. Four newly examined MN patients also exhibited reduced (gray arrowheads) or slightly elevated *IFI27* mRNA levels (gray arrows). Moreover, all of the patients with MGA, FSGS, and DN, as well as most of the patients with MCNS and BN, had reduced *IFI27* mRNA levels.

By contrast, *IFI27* mRNA levels in PBMCs were elevated in patients TN1–3, MK1, DIGI, MPGN1, LN1, LCDD1, and AN2 (white arrows in Fig 4B). Medical records revealed that patient DIGI1 patient had undergone IFN-β1b (Betaferon^®^) treatment for therapy of multiple sclerosis prior to blood collection; this treatment may induce *IFI27* expression. Inflammation was not directly involved because the C-reactive protein (CRP) and white blood cell (WBC) values were normal in other patients with elevated IFI27 expression; CRP <0.04 mg/dl except for patients LCDD1 (0.12) and TN1 (0.35); WBC, 4550–8930 cells/ml. Patient TN2 was undergoing treatment with soluble tumor necrosis factor-alpha (TNF-α)/lymphotoxin-alpha (LT-α) receptor for therapy of rheumatoid arthritis with Sjögren syndrome at the time of collection; *IFI27* expression could be indirectly induced by this treatment. Notably, patients MK1 (BJP type), LCDD1, and AN2 (IgG type) had been diagnosed with as multiple myeloma; TN1 also suffered from a fever from unknown origin; and LN1 also suffered from mixed connective tissue disease. These results suggest that the elevated *IFI27* mRNA levels in these cases were due to diseases other than CGN. Due to small size of the sample population for some of the diseases, additional studies with larger sample sizes will be needed to test the generalizability of our findings.

### Statistically significant reduction in *IFI27* mRNA level in PBMCs of IgAN patients

**Fig 5:**
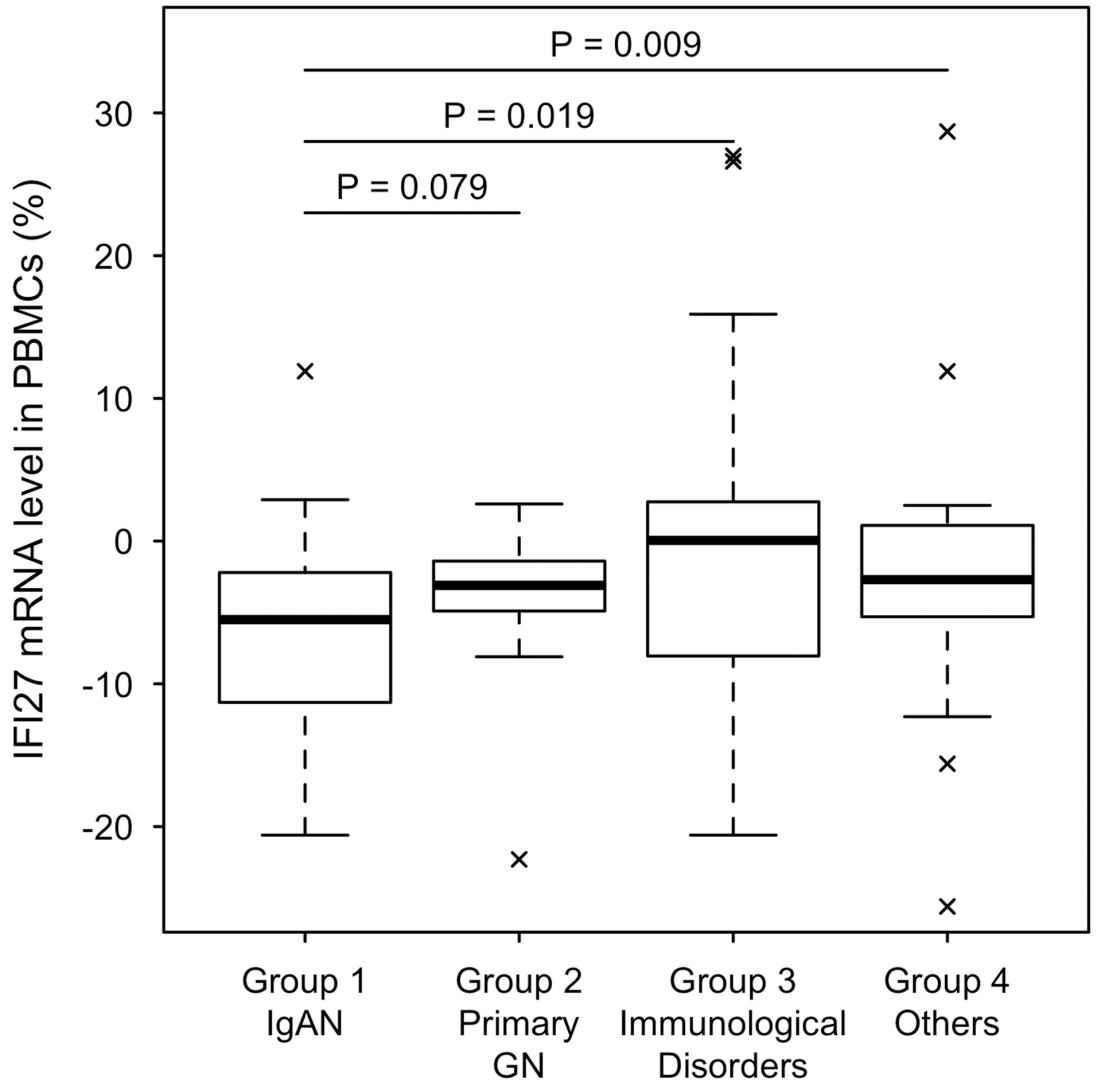
Statistical analysis of *IFI27* mRNA levels in PBMCs of various chronic glomerulonephritis patients, divided into four groups. Group 1: IgAN (n = 64), Group 2: Primary GN (n = 27), Group 3: Immunological Dis (n = 20), Group 4: Others (n = 34). TN2 and DIIG1 were plotted outside the figure because of their remarkably high *IFI27* mRNA levels (52.6% and 190.2%, respectively). IgAN, IgA nephropathy; Primary GN, primary glomerulonephritis; Immunological Dis, secondary glomerulonephritis due to immunological disorders; Others, other kidney diseases.

To explore the physiological significance of the reduced *IFI27* mRNA level in the analyzed patients, we performed the following statistical analysis (Fig 5). We first divided the patients into four groups. Group 1: IgAN (n = 64, 44.1%). Group 2: Primary glomerulonephritides (GN) (n = 27, 18.6%), including MN (n = 12), MCD (n = 11), and FSGS (n = 4). Group 3: Secondary glomerular diseases due to immunological disorders (n = 20, 13.8%), including LN (n = 2), IgAV (n = 4), AAG (n = 7), AGN (n = 1), MPA (n = 1), AN (n = 3), LCDD (n = 1), and MK (n = 1). Group 4: Other kidney diseases (n = 34, 23.4%), DN (n = 3), BN (n = 8), MPGN (n = 1), MH (n = 1), TM (n = 1), AS (n = 1), TBMD (n = 2), GSD (n = 1), LD (n = 1), SG (n = 2), TN (n = 6), DIIG (n = 1), NMT (n = 1), and MGA (n = 5). Patients TN2 and DIIG1 were plotted outside the figure because of their remarkably high *IFI27* mRNA levels (52.6% and 190.2%, respectively).

Median (25%, 75%) relative expression levels of *IFI27* (Fig 5) were -5.5% (-11.3, -2.2) for Group 1, -3.1 (-5.1, -1.5) for Group 2, 0.1 (-8.1, 2.8) for Group 3, and -2.6 (-.5.3, 1.1) for Group 4 (Kruskal—Wallis test, *P* = 0.0029). Secondary renal diseases due to immunological disorders and other diseases (Groups 3 and 4) were associated with higher *IFI27* mRNA level than IgAN. The Wilcoxon rank-sum test suggested that the statistical difference was either modest (*P* = 0.019) or clear (*P* = 0.009). Moreover, the *IFI27* mRNA level in primary GN (Group 2) was higher than that of IgAN at a marginally significant level (*P* = 0.079). These results suggest that reduced *IFI27* mRNA level is a useful marker that can be measured using RNA from PBMCs.

### IFI27 and WT1 immunohistochemical stainings

**Fig 6:**
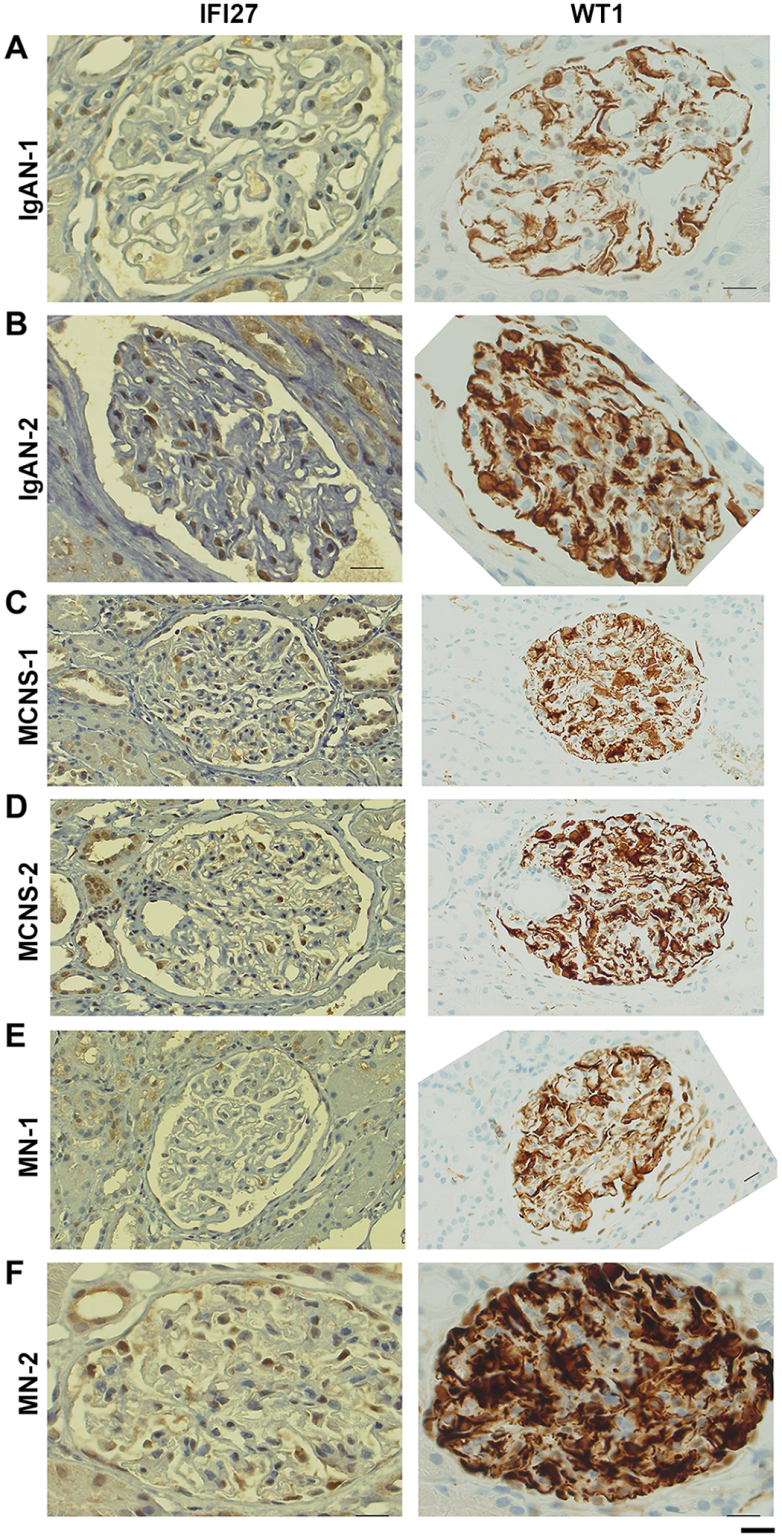
Distribution of IFI27-immunostained dots in the glomeruli of IgAN, MCNS, and MN patients. **(A–F)** Typical images of IFI27 immunohistochemistry (160×) in renal specimens from two IgAN, two MCNS, and two MN patients. Gender and age of these patients are shown in S4D Fig. Bar, 20 μm.

IFI27 is preferentially expressed in glomeruli, as determined by qRT-PCR; more precisely, as determined by *in situ* hybridization [27], *IFI27* mRNA is detected preferentially in glomerular podocytes. To determine whether expression of IFI27 protein is also reduced in patients with kidney disease, we performed IHC with an anti-IFI27 antibody on biopsied glomeruli samples from IgAN, MN, and MCNS patients. We also performed IHC using an antibody against Wilms tumor 1 (WT1) protein that is highly expressed in podocyte and has been used as a podocyte marker [28]. Indeed, only a small number of IFI27 positive signals were observed in the glomeruli of IgAN-1, IgAN-2 and MN-1 patients, whereas WT1 positive signals were detected throughout the glomeruli, suggesting the abundant population of podocytes with low IFI27 expressions in the glomeruli of these patients (Fig 6A, 6B and 6E). MCNS-1, MCNS-2 and MN-2 patients showed comparatively lots of IFI27 positive signals, but their signal intensities were weak and they are restricted into nucleus (Fig 6C, 6D and 6F). By contrast, WT1 positive signals were strong and observed in both cytoplasm and nucleus of podocytes (Fig 6). Other images from different samples also showed a low abundance of IFI27 positive signals in the podocytes of IgAN, MCNS and MN patients (S5–S10 Figs). By contrast, WT1 positive signals were strongly detected in the podocytes of these patients (S11–S13 Figs). These results are consistent with the fact that the *IFI27* mRNA levels in most IgAN, MCNS and MN patients (Fig 4). Unfortunately, glomeruli samples from IFI27-elevated patients were not available. Overall, these results suggest that the IFI27 protein level is reduced in IgAN, MCNS and MN patients.

## Discussion

In this study, we performed DNA microarray analysis and identified several genes that were up- or down-regulated in PBMCs of 15 IgAN patients and eight MN patients relative to HVs (Fig 1 and S3 Fig). Scatter plot analysis of the high-ranked genes (Fig 2) was useful for determining whether they were physiologically significant or not. Note that a 5-fold change (45 versus 9) in the case of KRTAP8-1 (IgAN/MN versus HVs) could not be considered to be physiologically significant when the measurement error was 10. By contrast, a 20-fold change (8,000 versus 400) in the case of IFI27 (HVs versus IgAN/MN) was physiologically significant (Fig 2). Indeed, qRT-PCR for *KRTAP8-1* failed to validate the DNA microarray results (S2F Fig). We also performed qRT-PCR analyses on other clinically noteworthy genes such as *CLSTN1*, *COTL1* and *KRTAP5-8*, and found that they also failed to reproduce the DNA microarray data (S2 Fig). By contrast, *IFI27* mRNA levels exhibited similar reductions between the DNA microarray and qRT-PCR analyses (Fig 3). We also found that *IFI27* mRNA levels were reduced in the PBMCs of patients with other kidney diseases, although the reductions were less prominent than those in IgAN and MN (Fig 4). Statistical analyses confirmed that *IFI27* mRNA levels were more conspicuously reduced in IgAN than in other kidney diseases (Fig 5). IHC analysis also showed that IFI27 protein level was reduced in glomeruli in biopsied samples from IgAN and MN patients (Fig 6). These results suggest that IFI27 is potentially a useful genetic marker for diagnosis of IgAN and MN using peripheral blood. Note that the age and gender of the patients (S1 and S4 Figs) affect neither the *IFI27* mRNA levels (Figs 3 and 4) or the IHC results (Fig 6 and S5–S9 Figs).

IFI27 is an interferon-α inducible protein that is up-regulated in PBMCs of systemic lupus erythematosus patients [23] and in the lung after influenza A infection in mice, mainly due to the infiltration of macrophages and lymphocytes [29]. It is also up-regulated in inflammatory psoriatic skin and in some epithelial cancers [24], such as ovarian cancer, and its expression is associated with patient survival [30]. In a mouse model, IFI27 knockdown in keratinocytes slows their proliferation and decreases epidermal thickness and formation of rete ridges in imiquimod-induced psoriatic lesions [31]. Thus, up-regulation of IFI27 may be involved in inflammatory events. By contrast, IFI27 is down-regulated and exhibits a reduced response to IFNα stimulation in uterine leiomyomas relative to myometrial cells [32]. IFI27/ISG12 is a novel modulator of innate immune responses that regulate anti-inflammatory nuclear receptors, and experiments using IFI27/ISG12 deficient mice revealed that a lack of ISG12 prolongs survival in experimental sepsis and endotoxemia [33]. As described above, *IFI27* is preferentially expressed in glomeruli and IFI27 is abundant in podocytes [27]. These observations suggest that IFI27 is involved in the regulation of inflammatory events in PBMCs and podocytes. Because IFI27/ISG12 deficient mice show no abnormality in kidney functions, it remains unclear how these IFI27-related events are involved in the pathgenesis of kidney diseases such as IgAN and MN. Further studies will be required to address this issue.

IFI27 levels in IgAN patients were significantly lower than those in patients with other kidney diseases (Figs 4 and 5). IgAN is thought to be induced by oral or tonsillar bacterial infection; consistent with this, specific bacterial infections have been detected in specimens obtained by IgAN tonsillectomy, a useful standard therapy for IgAN [11], and other groups also reported a relationship between oral bacterial infections and IgAN [10], [34]. Notably, highly sensitive CRP is not a useful indicator of these infections, probably because they are limited to the tonsillar areas [35]. Toll-like receptor 9 (TLR9) is expressed at higher levels in both IgAN model mice [36] and IgAN patients [37], perhaps because a variable copy-number region at 3p21.1 influences TLR9 expression levels in IgAN patients [38]. Indeed, repeated stimulation of TLR9 inhibits IFN-alpha production [39]. Thus, continuous bacterial infection in the restricted region might suppress IFN-alpha production, resulting in a decrease in IFI27 levels. MN is also associated with bacterial infection [40]. Therefore, the IFI27 level in MN patients might be influenced by such infections. Although little is known about the physiological roles of IFI27 in podocyte, our results suggest that IFI27 might also be important for normal podocyte function after viral attack, and that glomeruli in which *IFI27* is persistently down-regulated by continuous bacterial infection might be vulnerable to kidney injury from CGN, in particular IgAN and MN. Thus, a more reliable clinical marker is required for chronic kidney diseases, including primary glomerulonephritis. Our results suggest that a lower level of IFI27 in PBMCs might be a good marker for CGN, especially IgAN, the most common primary glomerulonephritis.

This study had several limitations. First, our results must be confirmed in other cohorts before they are applied to clinical use. Second, although we evaluated the IFI27 levels in many other chronic kidney diseases, including MK and TN (Fig 4), the distributions of IFI27 levels in these diseases were not well defined because of the small number of patients analyzed. Therefore, the distribution of IFI27 levels in these diseases should be confirmed in future studies. Third, given our results suggesting that interferon treatment increased IFI27 expression, viral infection might have some influence on IFI27 level (Fig 4C, case DIGI1). Therefore, prior to clinical applications of IFI27 levels, they should be evaluated in the context of viral infections. Fourth, we did not evaluate IFI27 levels after treatment. After IgAN tonsillectomy, which should eliminate bacterial infection of the tonsils, IFI27 levels might recover to normal levels; consistent with this, the IFI27 level was normal in an IgAN patient who had previously undergone tonsillectomy (Fig 4A, patient IgAN25). Therefore, future studies should monitor IFI27 levels over a time course after treatment.

## Conclusion

Based on our results, we propose that IFI27 may serve a useful marker for diagnosing CGN using peripheral blood.

## Materials and Methods

### Human subjects and ethical considerations

A total of 137 sequential patients who underwent kidney biopsy and had blood samples collected during February 2009 to March 2012 at Osaka University Hospital (Hospital A) were enrolled in this study at the time of examination. Blood samples were also collected from March 2008 to June 2008 from eight IgAN patients at Kitano Hospital (Hospital B). Those patients had been comprehensively diagnosed by kidney biopsy, medical history, and blood examinations. Samples from the first 15 IgAN and eight MN patients were subjected to DNA microarray analysis, and the other samples were subjected to extended qRT-PCR analysis. The age and gender of 17 HVs are shown in our previous report [19]; the principle of pooling of HV sample is to compare the results with those of our previous study [18–20]. The study was reviewed and approved by the Research Ethics Committee of Osaka University and Kitano Hospital. Written informed consent was obtained from all participants when serum samples were obtained.

### RNA isolation

Total RNA isolation of leukocytes from whole blood of 15 IgAN and eight MN patients was performed using the PAXgene^™^ Blood RNA System (BD Bioscience, San Jose, CA, USA). The integrity of total RNA used for microarray analysis was confirmed using the RNA 6000 Nano LabChip Kit (p/n 5067–1511) on an Agilent 2100 Bioanalyzer (G2938C; Agilent Technologies Inc., Palo Alto, CA, USA), and only samples with an RNA integrity number (RIN) above 7.4 were used for gene expression profiling.

### DNA microarray analysis

Total RNA (500 ng) isolated from the PBMCs of 15 IgAN and eight MN patients was examined along with pooled RNA from healthy volunteers (S1 Fig). RNA from each patient and the pooled normal RNA were reverse-transcribed with oligo-dT primers containing the T7 RNA polymerase promoter sequence. The resulting cDNA was subjected to *in vitro* transcription with T7 RNA polymerase for Cy3 labeling (CyDye; Amersham Pharmacia Biotech). Cy3-labeled cRNAs from the 15 IgAN and eight MN patients (825 ng) were hybridized on Agilent Whole Human Genome Microarrays (4x44K G4112F). The signal intensity of Cy3 was calculated for every probe, and the results were analyzed with the Subio Basic Plug-in (v1.6; Subio Inc.), which allows the visualization of microarray data in the form of a heat map and a line graph. The microarray data have been deposited in the Gene Expression Omnibus (GEO; www.ncbi.nlm.nih.gov/geo) database (accession number: GSE73953). Agilent Feature Extraction software (v. 9.5.1) was used to assess spot quality and extract feature intensity statistics. The Subio Platform and Subio Basic Plug-in (v1.11; Subio Inc., Aichi, Japan) were then used to calculate the between-sample *fold change*.

### Expression profiling using qRT-PCR

ABI PRISM 7900 (PE Applied Biosystems, Foster City, CA) was used to perform qRT-PCR using the Assay-on-Demand TaqMan probe and gene-specific primers. Total RNA (500 ng) was reverse-transcribed using the High Capacity cDNA Archive Kit (ABI). The resultant cDNA was used as a template for PCR in a 20 μL reaction containing 10 μl of 2× Master Mix (TaKaRa, Otsu, Japan). Assay kits for *IFI27*, *CLSTN*, *KRTAP8-1* and *KRTAP5-8* (ID Hs01086373_g1, Hs00208929_m1, Hs00545666_s1 and Hs03037928_g1, respectively) were purchased from PE Applied Biosystems. As for *GAPDH*, the following oligonucleotides were used as primers and probes: *GAPDH* forward primer, 5'-CCATCAATGACCCCTTCATTG-3'; *GAPDH* reverse primer, 5'-TCTCGCTCCTGGAAGATGGT-3'; and *GAPDH* TaqMan probe, 5'-VIC-ACCTCAACTACATGGTTTAC-MGBNFQ-3'. PCR reactions were conducted on the following conditions (each sample was assayed in quadruplicate): initial denaturation at 95°C for 10 min, followed by 40 cycles of denaturation at 95°C for 15 s and annealing/extension at 60°C for 1 min. The median threshold cycle (C_T_) values were used to calculate fold changes between the treated and control samples, and a standard curve was generated from the amplification data for each primer using serial dilutions of PBMC RNA as the template. Fold change values were normalized to the corresponding levels of *GAPDH* levels using the standard curve method.

### IFI27 immunohistochemical staining

Kidney biopsy samples from biopsy-proven two IgAN, two MCNS and two MN patients were stained using anti-IFI27 primary antibody (Abcam, UK). Patient backgrounds are summarized in S4D Fig. IFI27 immunohistochemical staining using kidney biopsy samples was performed as follows. First, paraffin-embedded tissue samples from consenting patients were cut in 5 μm sections. The samples were then deparaffinized in xylene and rehydrated using a series of graded alcohols. Antigen retrieval was performed by heating in 20 mM citrate buffer (pH 6). Samples were blocked with 10% goat serum and then incubated overnight with anti-IFI27 primary antibody (1:200 dilution) in a humidified container at 4°C. Immunohistochemical staining was performed using the Dako Envision Plus System (Dako, Carpinteria, CA, USA).

### WT1 immunohistochemical staining

We performed WT1 1HC of kidney biopsy samples using a serial section used for IFI27 IHC as follows. Firstly, the paraffin-embedded tissue samples from patients with informed consent were cut in 5-micrometer sections. Then, the samples were deparaffinized in xylene and rehydrated using a series of graded alcohols. Antigen retrieval was performed by heat mediation in 20 mM citrate buffer (pH 6). Samples were blocked with 10% goat serum before incubating with the primary antibody. The samples were incubated at 1:50 dilution overnight using an anti-WT1 antibody (Agilent's Dako, Glostrup, Denmark) in a humidified container at 4°C. IHC results were visualized using Dako Envision Plus System (Dako, Carpinteria, CA) according to the manufacturer’s instructions.

### Statistical analyses

The mean expression levels of *IFI27* across four groups were compared using the Kruskal—Wallis test. The expression levels in primary glomerulonephritides, secondary renal diseases due to immunological disorders, and other diseases were compared with the level in IgAN using the Wilcoxon rank-sum test. P < 0.05 was considered statistically significant. All statistical analyses were performed using STATA, version 14 (STATA Corp, www.stata.com).

## Supporting Information

S1 Fig Gender and age distributions of IgAN and MN patients. RNA purified from PBMCs of these patients was subjected to DNA microarray analysis. Females and males are indicated by circles and triangles, respectively.
(TIF)

S2 Fig Comparison of DNA microarray and qRT-PCR data. Relative mRNA levels, determined by DNA microarray **(A, C, E, G)** or qRT-PCR **(B, D, F, H)**, are shown as box graphs for *CLSTN* **(A, B)**, *COTL1* **(C, D)**, *KRTAP8-1* **(E, F)**, and *KRTAP5-8* **(G, H)** using RNA samples from individual IgAN and MN patients. Vertical axis indicates the mRNA level (arbitrary units: a.u.) relative to the value in HVs, which was fixed at 1.0 a.u.
(TIF)

S3 Fig Expression profiles of genes in PBMCs of 15 IgAN and eight MN patients. **(A)** List of the top 31 genes up-regulated in most IgAN patients (fold-change >3.0), but not in MN patients (fold-change <1.1), shown in decreasing order of fold-change values for IgAN. *KRTAP5-8* (red font) was subjected to qRT-PCR analysis (see S2 Fig). **(B)** List of the bottom 22 genes down-regulated in most IgAN patients (fold-change <1.1), but not in MN patients (fold-change >3.0), shown in increasing order of fold-change values for IgAN. “Unknown” indicates uncharacterized genes. Mosaic tile representation for each gene is also shown, with intensity gradients indicating the mean value of the expression level (log_2_ ratio): down-regulation (green) and up-regulation (red) are expressed relative to the average value in healthy volunteers (yellow). **(C)** Bar represents the standard intensity gradient.
(TIF)

S4 Fig List of patients subjected to qRT-PCR analysis. **(A–C)** Patients correspond to those in Fig 4. Sample code number, abbreviated symbol, full description of disease names, fold-change values, age, and gender of each patient are shown.
(TIF)

S5 Fig IFI27 immunostaining of the glomeruli from patient IgAN-1. **(A-F)** Typical images of IFI27 immunohistochemistry for six pairs of independent glomeruli from the same biopsied specimens. Enlarged views of the above images are shown at the bottom of each image. Bar, 20 μm.
(TIF)

S6 Fig IFI27 immunostaining of glomeruli from patient IgAN-2. **(A-F)** Typical images of IFI27 immunohistochemistry for six pairs of independent glomeruli from the same biopsied specimens. Enlarged views of the above images are shown at the bottom of each image. Bar, 20 μm.
(TIF)

S7 Fig IFI27–immunostaining of the glomeruli from patient MCNS-1. **(A-F)** Typical images of IFI27 immunohistochemistry for six pairs of independent glomeruli from the same biopsied specimens. Enlarged views of the above images are shown at the bottom of each image. Bar, 20 μm.
(TIF)

S8 Fig IFI27 immunostaining of glomeruli from patient MCNS-2. **(A-F)** Typical images of IFI27 immunohistochemistry for four independent glomeruli from the same biopsied specimens. Enlarged views of the region encircled by red or turquoise lines are shown at the bottom of each image. Bar, 20 μm.
(TIF)

S9 Fig IFI27 immunostaining of glomeruli from patient MN-1. **(A-F)** Typical images of IFI27 immunohistochemistry for six pairs of independent glomeruli from the same biopsied specimens. Enlarged views of the above images are shown at the bottom of each image. Bar, 20 μm.
(TIF)

S10 Fig IFI27 immunostaining of glomeruli from patient MN-2. **(A-F)** Typical images of IFI27 immunohistochemistry for six pairs of independent glomeruli from the same biopsied specimens. Enlarged views of the above images are shown at the bottom of each image. Bar, 20 μm.
(TIF)

S11 Fig WT1 immunostaining of the glomeruli from patient IgAN-1 (A-F) and IgAN-2 (G-L). Typical images of WT1 immunohistochemistry for 12 independent glomeruli from biopsied specimens of two IgAN patients, IgAN-1 (A-F) and IgAN-2 (G-L). Bar, 20 μm.
(TIF)

S12 Fig WT1 immunostaining of the glomeruli from patient MCNS-1 (A-F) and MCNS-2 (G-L). Typical images of WT1 immunohistochemistry for 12 independent glomeruli from biopsied specimens of two MCNS patients, MCNS-1 (A-F) and MCNS-2 (G-L). Bar, 20 μm.
(TIF)

S13 Fig WT1 immunostaining of the glomeruli from patient MN-1 (A-F) and MN-2 (G-L). Typical images of WT1 immunohistochemistry for 12 independent glomeruli from biopsied specimens of two MN patients, MN-1 (A-F) and MN-2 (G-L). Bar, 20 μm.
(TIF)

S14 Fig Expression profiling of TNFSF genes. **(A)** Mosaic tile representation of TNFSF genes for HV1, HV2, and 15 IgAN patients. **(B)** Mosaic tile representation of TNFSF genes for eight MN patients. Intensity gradients indicate the mean value of the expression level (log2 ratio): down-regulation (green) and up-regulation (red) are shown relative to the average value in healthy volunteers (yellow). Arrows highlight the indicated genes.
(TIF)

S15 Fig Gene map and expression profiling of genes in the vicinity of TNFSF12 and TNFSF13. **(A)** Distribution of the genes near TNFSF12 and TNFSF13: this region on chromosome 17 exhibits naturally occurring read-through transcription. POLR2A: polymerase (RNA) II (DNA-directed) polypeptide A; E1F4A1: eukaryotic initiation factor 4A1; SENP3:SUMO1/sentrin/SMT3 specific peptidase 3. **(B)** Mosaic tile representation of TNFSF12, TNFSF13, E1F4A1, and SENP3 for HV1, HV2, and 15 IgAN and eight MN patients. Intensity gradients indicate the mean value of the expression level (log2 ratio): down-regulation (green) and up-regulation (red) are shown relative to the average value in healthy volunteers (yellow). Arrows highlight notable genes.
(TIF)

S16 Fig Expression profiling of complement factor-related genes. **(A)** Mosaic tile representation of complement factor-related genes for HV1, HV2, and 15 IgAN patients. **(B)** Mosaic tile representation of TNFSF genes for eight MN patients. Intensity gradients indicate the mean value of the expression level (log2 ratio): down-regulation (green) and up-regulation (red) are shown relative to the average value in healthy volunteers (yellow). Arrows highlight notable genes.
(TIF)

S17 Fig Scatter plot of DNA microarray data obtained from IgAN patients from two independent hospitals. **(A)** Scatter plot over the entire signal intensity range to show that the expression arrays we used here provide a high-resolution platform. **(B)** Comparison of fold change over the entire analyzed genes to assess the frequency of commonly up-or down-regulated mRNA levels between IgAN patients of these two independent hospitals.
(TIF)

S18 Fig Profiles of mRNA levels in PBMCs of IgAN patients from two independent hospitals. Line-graphs that represent 5 genes (in red font) whose mRNA levels were up-regulated in almost all IgAN patients from Osaka Univ. Hospital **(A)** and Kitano Hospital **(B)**. Intensity gradients indicate the mean value of the mRNA level (log2 ratio): down-regulation (green) and up-regulation (red) are shown relative to the average value in HVs (yellow).
(TIF)

S1 Text (Supplementary Results) Altered mRNA levels of *TNFSF* and complement factor genes in IgAN and MN patients. (DOCX)

## Abbreviations

AS: Alport syndrome
AN: amyloid nephropathy
AGN: anti-GBM glomerulonephritis
ANCA: anti-neutrophil cytoplasmic antibody
AAG: ANCA-associated glomerulonephritis
BN: benign nephrosclerosis
CFH: complement factor h
DN: diabetic nephropathy
DIIG: drug-induced glomerular injury
E1F4A: eukaryotic initiation factor 4A
FSGS: focal segmental glomerulosclerosis
GWAS: genome-wide association study
GSD: glycogen storage diseas
IgAN: immunoglobulin A nephropathy
IgAV: IgA vasculitis
IHC: immunohistochemistry
ISH: *in situ* hybridization
IFN: interferon
IFI27: IFN-alpha-inducible protein 27 gene
ISG12: IFN-stimulated gene 12
LCDD: light chain deposition disease
LCAT: lecithin: cholesterol acyltransferase
LCAD: LCAT deficiency
LN: lupus nephritis
LT-α: lymphotoxin-alpha
MN: membranous nephropathy
MPA: microscopic polyangitis
MCNS: minimal change nephrotic syndrome
MGA: minor glomerular abnormalities
MK: myeloma kidney
NMT: nephritis after bone marrow transplantation
PBMCs: peripheral blood mononuclear cells
POLR2A: polymerase (RNA) II (DNA-directed) polypeptide A
qRT-PCR: quantitative real-time reverse transcription polymerase chain reaction
*SENP3*: SUMO1/sentrin/SMT3 specific peptidase 3
TBM: thin basement membrane disease
TMA: thrombotic microangiopathy
TN: tubulointersitial nephritis
TNF-α: tumor necrosis factor-alpha
WT1: Wilms Tumor 1
TNFSF13: tumor necrosis factor ligand superfamily member 13
