# Supplementary figures and images for: IFI27 Is a Useful Genetic Marker for Diagnosis of Immunoglobulin A Nephropathy and Membranous Nephropathy Using Peripheral Blood
Source: PLoS One. 2016 Apr 21;11(4):e0153252. doi: 10.1371/journal.pone.0153252 (PMC4839700; doi:10.1371/journal.pone.0153252)

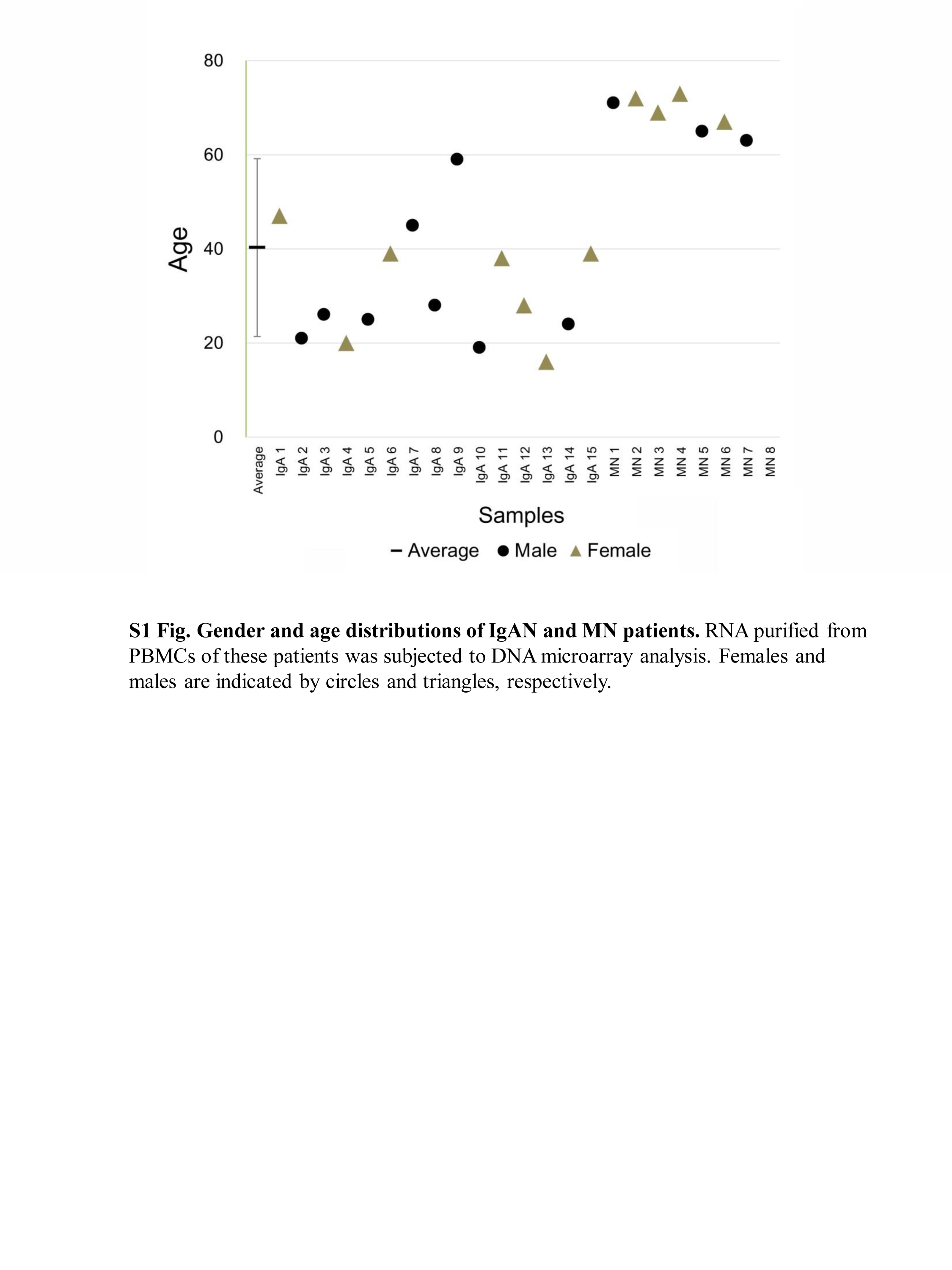

Supplement: S1 Fig — RNA purified from PBMCs of these patients was subjected to DNA microarray analysis. Females and males are indicated by circles and triangles, respectively. (TIF) [file pone.0153252.s001.tif]

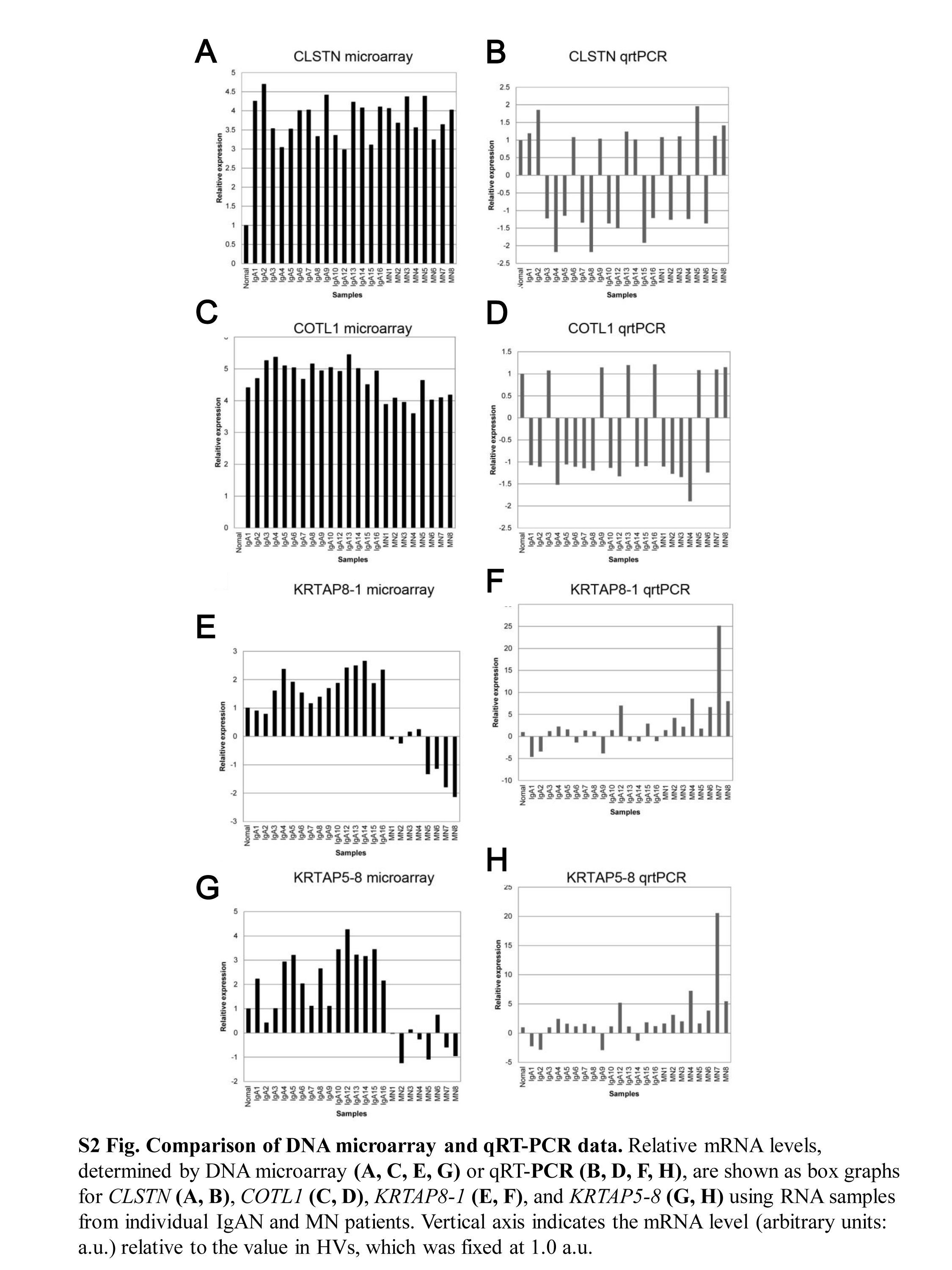

Supplement: S2 Fig — Relative mRNA levels, determined by DNA microarray (A, C, E, G) or qRT-PCR (B, D, F, H), are shown as box graphs for CLSTN (A, B), COTL1 (C, D), KRTAP8-1 (E, F), and KRTAP5-8 (G, H) using RNA samples from individual IgAN and MN patients. Vertical axis indicates the mRNA level (arbitrary units: a.u.) relative to the value in HVs, which was fixed at 1.0 a.u. (TIF) [file pone.0153252.s002.tif]

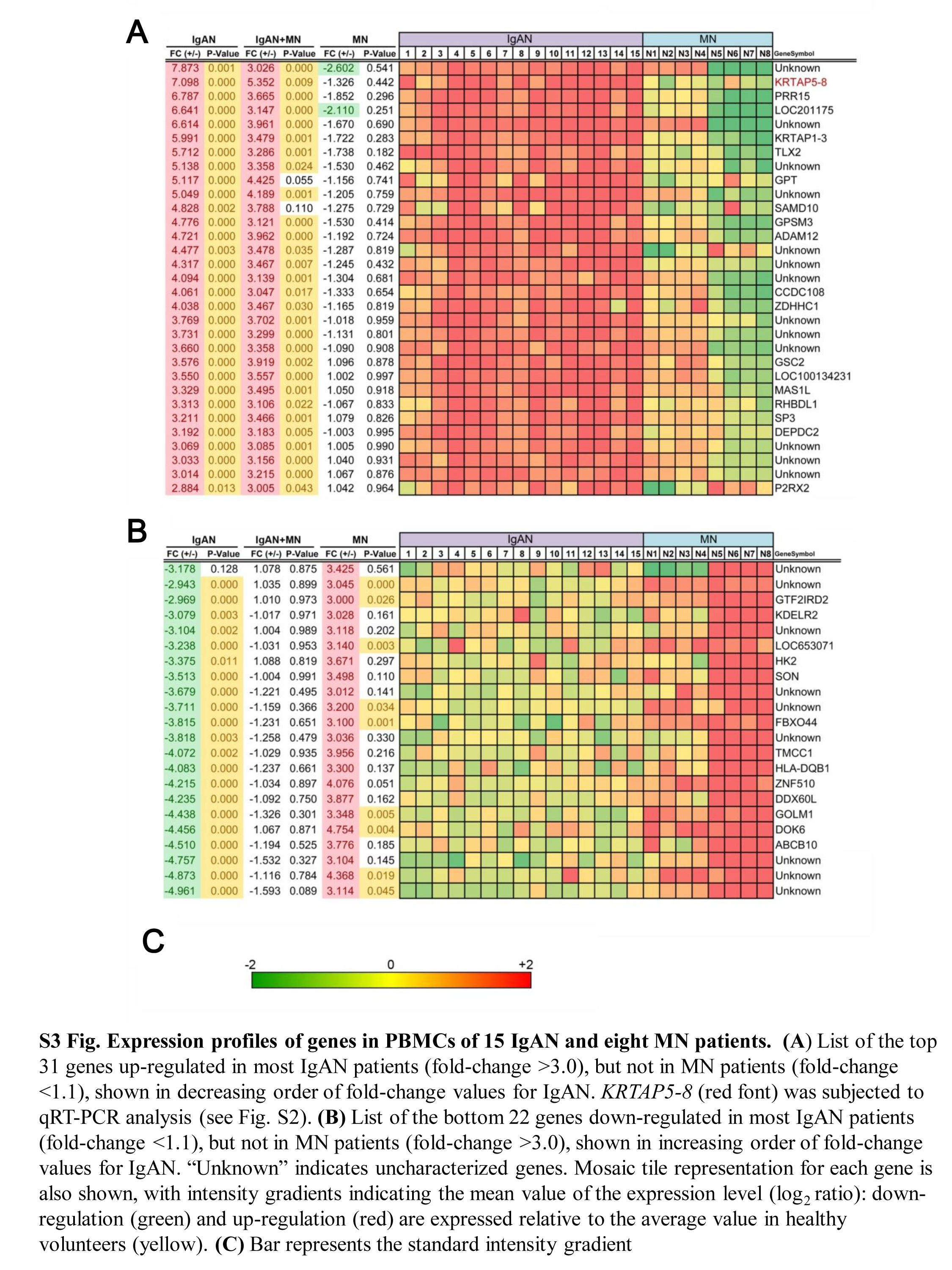

Supplement: S3 Fig — (A) List of the top 31 genes up-regulated in most IgAN patients (fold-change >3.0), but not in MN patients (fold-change <1.1), shown in decreasing order of fold-change values for IgAN. KRTAP5-8 (red font) was subjected to qRT-PCR analysis (see S2 Fig). (B) List of the bottom 22 genes down-regulated in most IgAN patients (fold-change <1.1), but not in MN patients (fold-change >3.0), shown in increasing order of fold-change values for IgAN. “Unknown” indicates uncharacterized genes. Mosaic tile representation for each gene is also shown, with intensity gradients indicating the mean value of the expression level (log2 ratio): down-regulation (green) and up-regulation (red) are expressed relative to the average value in healthy volunteers (yellow). (C) Bar represents the standard intensity gradient. (TIF) [file pone.0153252.s003.tif]

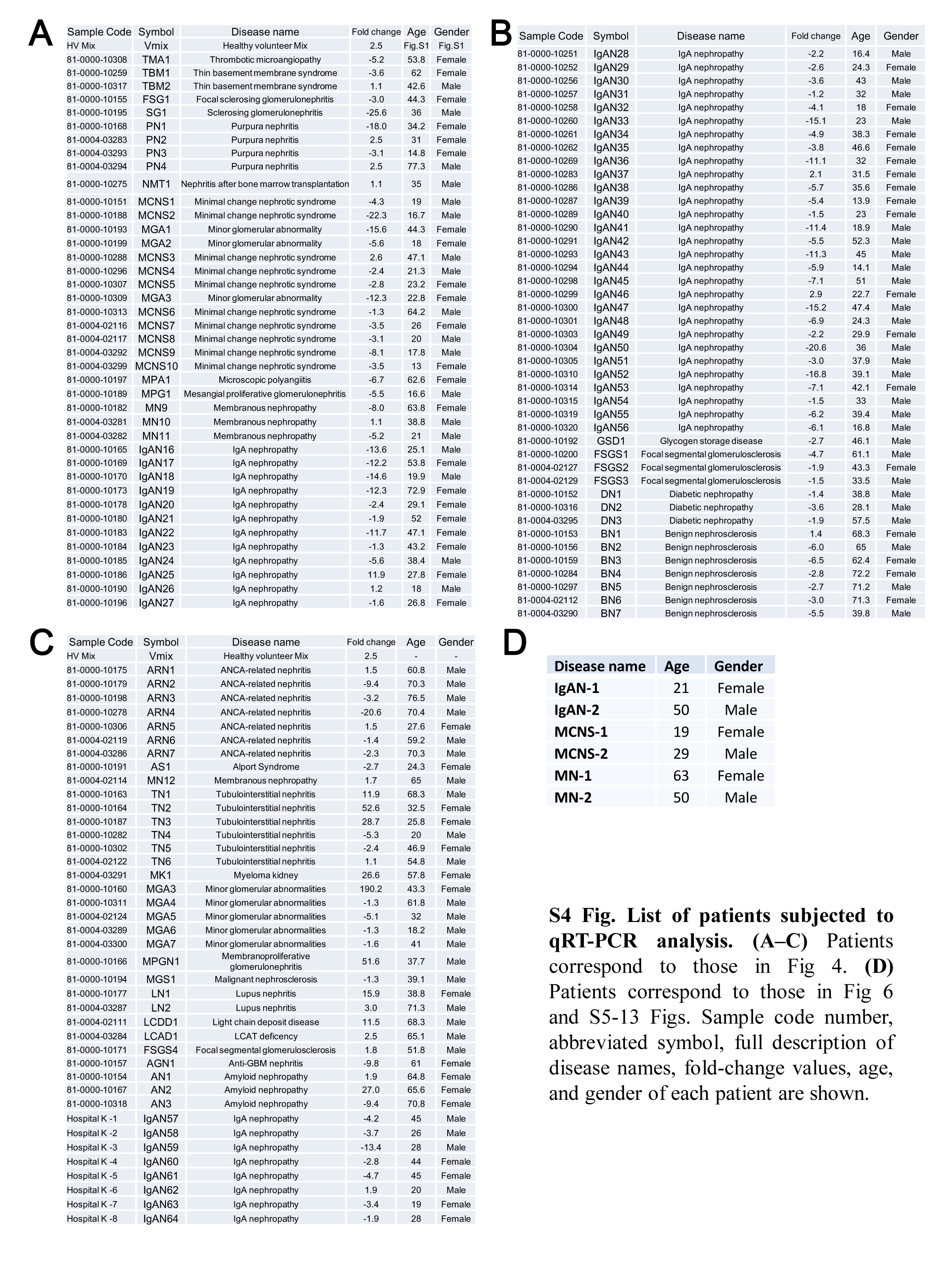

Supplement: S4 Fig — (A–C) Patients correspond to those in Fig 4. Sample code number, abbreviated symbol, full description of disease names, fold-change values, age, and gender of each patient are shown. (TIF) [file pone.0153252.s004.tif]

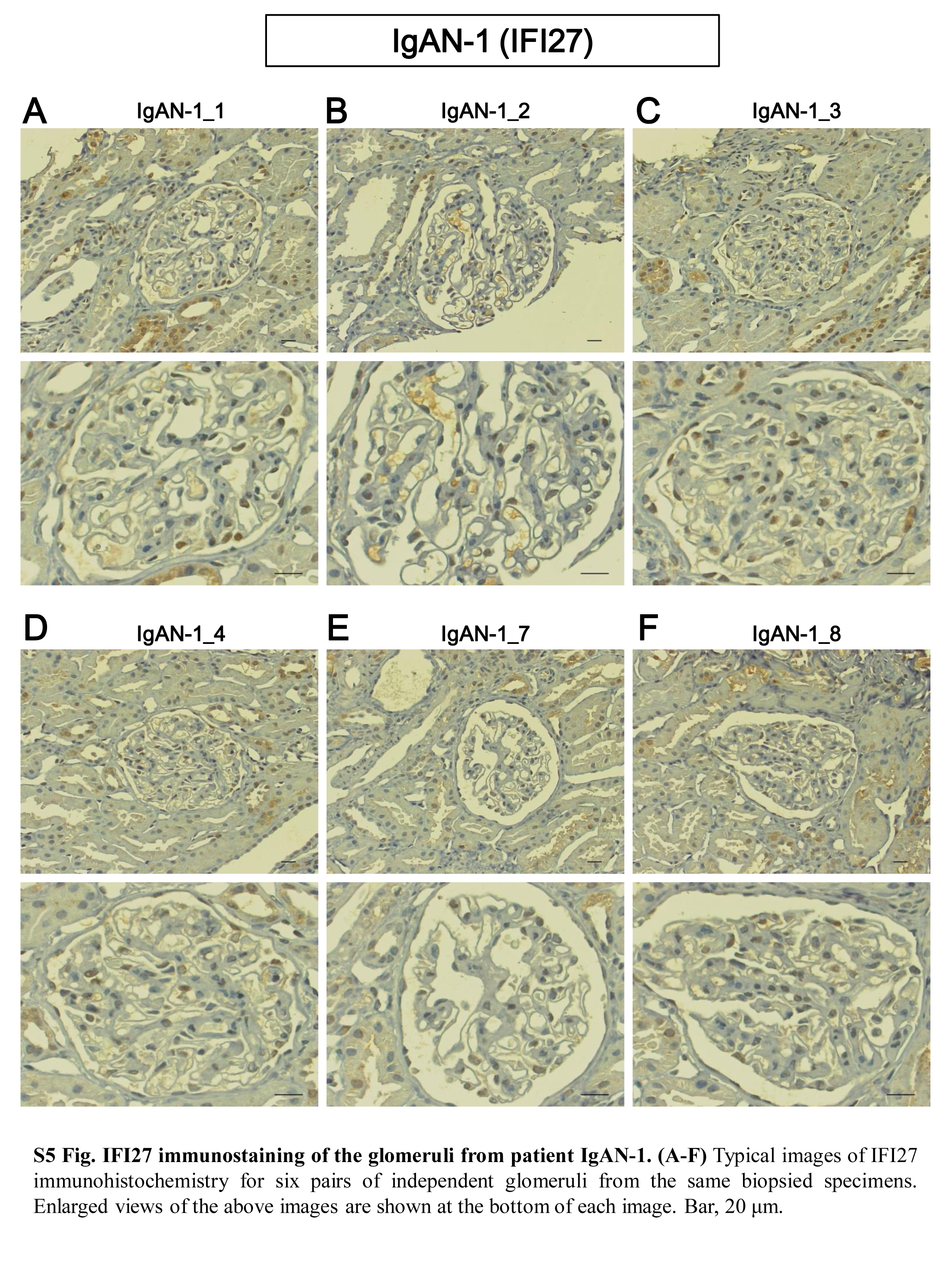

Supplement: S5 Fig — (A-F) Typical images of IFI27 immunohistochemistry for six pairs of independent glomeruli from the same biopsied specimens. Enlarged views of the above images are shown at the bottom of each image. Bar, 20 μm. (TIF) [file pone.0153252.s005.tif]

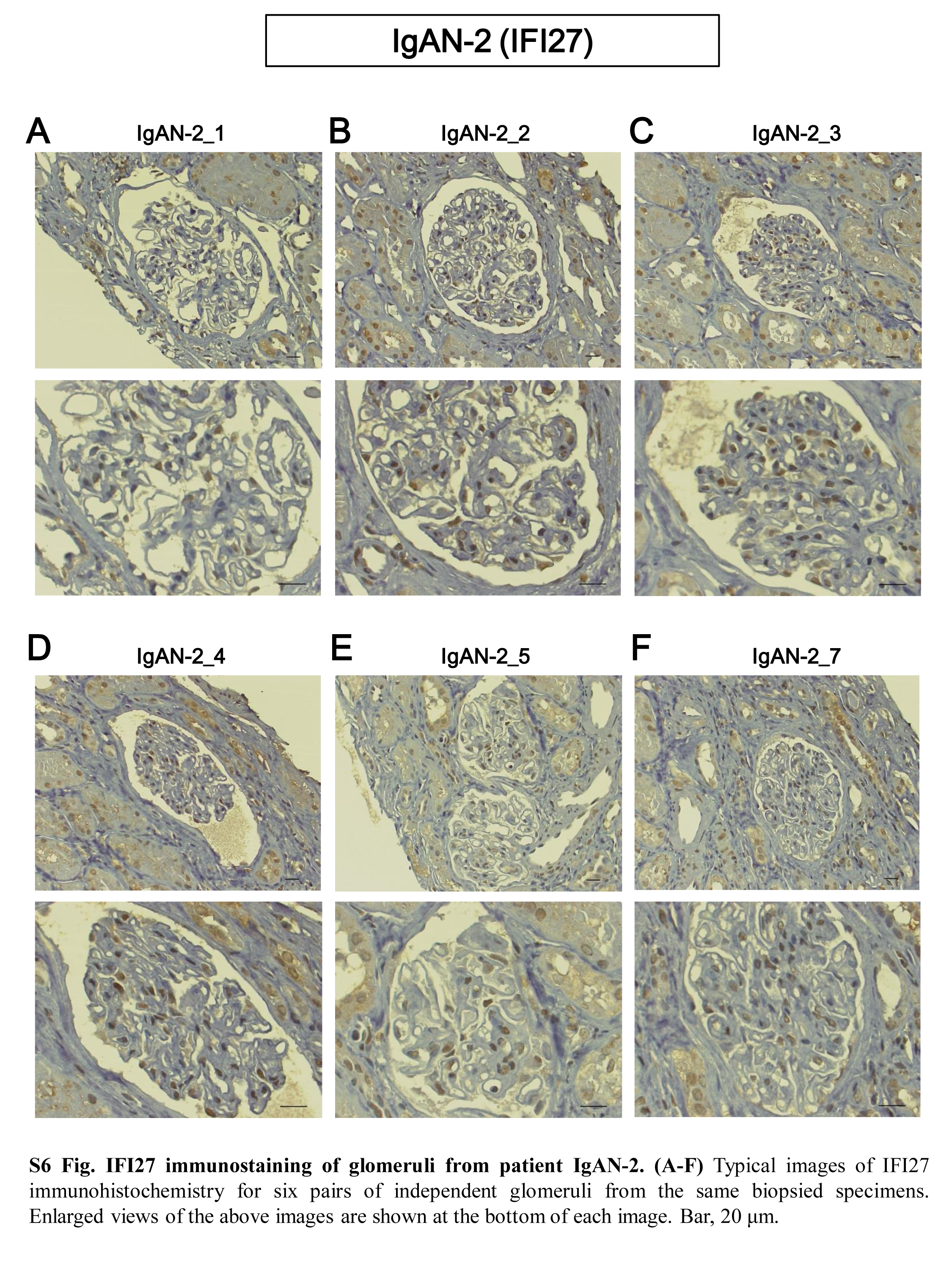

Supplement: S6 Fig — (A-F) Typical images of IFI27 immunohistochemistry for six pairs of independent glomeruli from the same biopsied specimens. Enlarged views of the above images are shown at the bottom of each image. Bar, 20 μm. (TIF) [file pone.0153252.s006.tif]

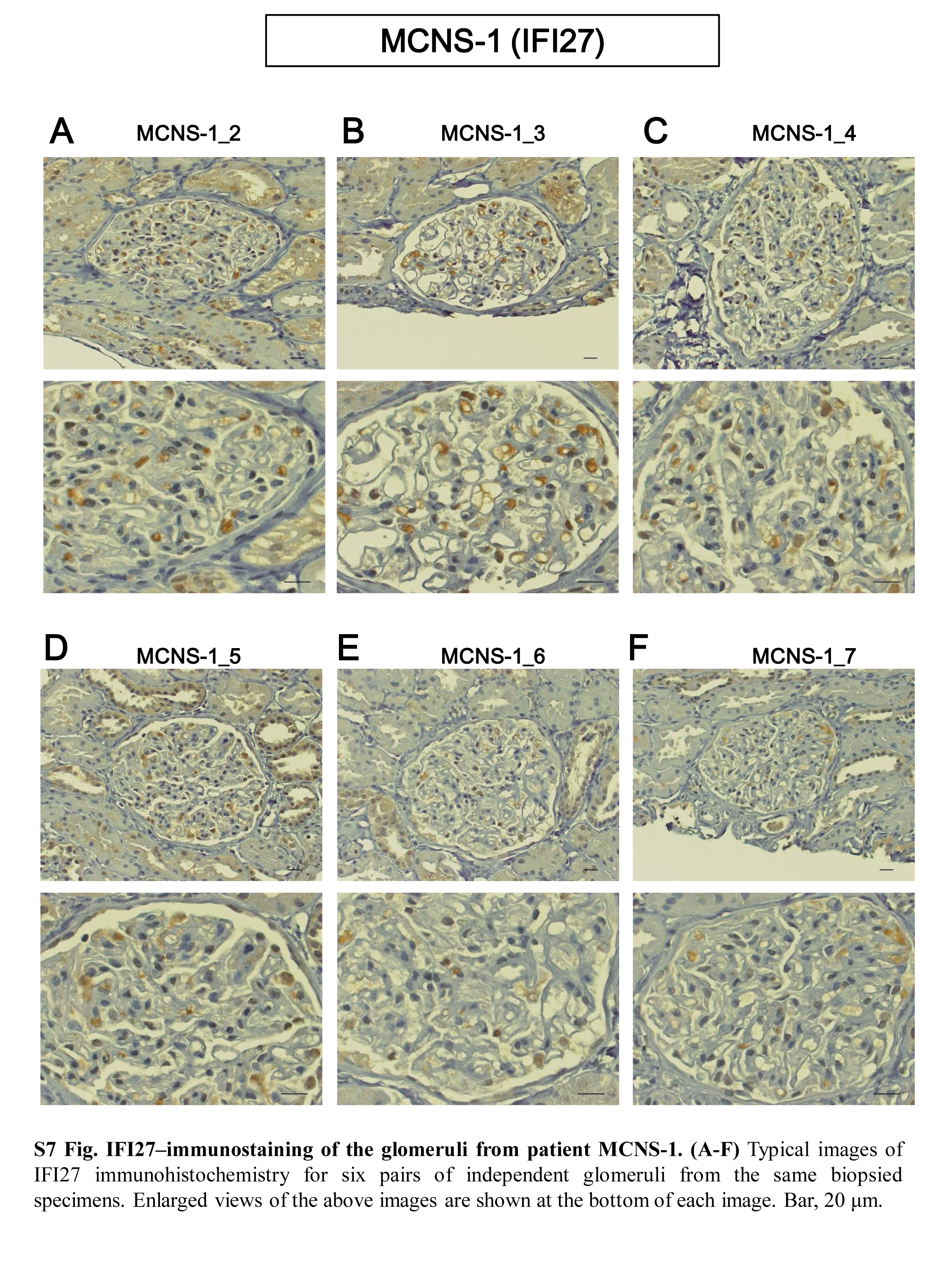

Supplement: S7 Fig — (A-F) Typical images of IFI27 immunohistochemistry for six pairs of independent glomeruli from the same biopsied specimens. Enlarged views of the above images are shown at the bottom of each image. Bar, 20 μm. (TIF) [file pone.0153252.s007.tif]

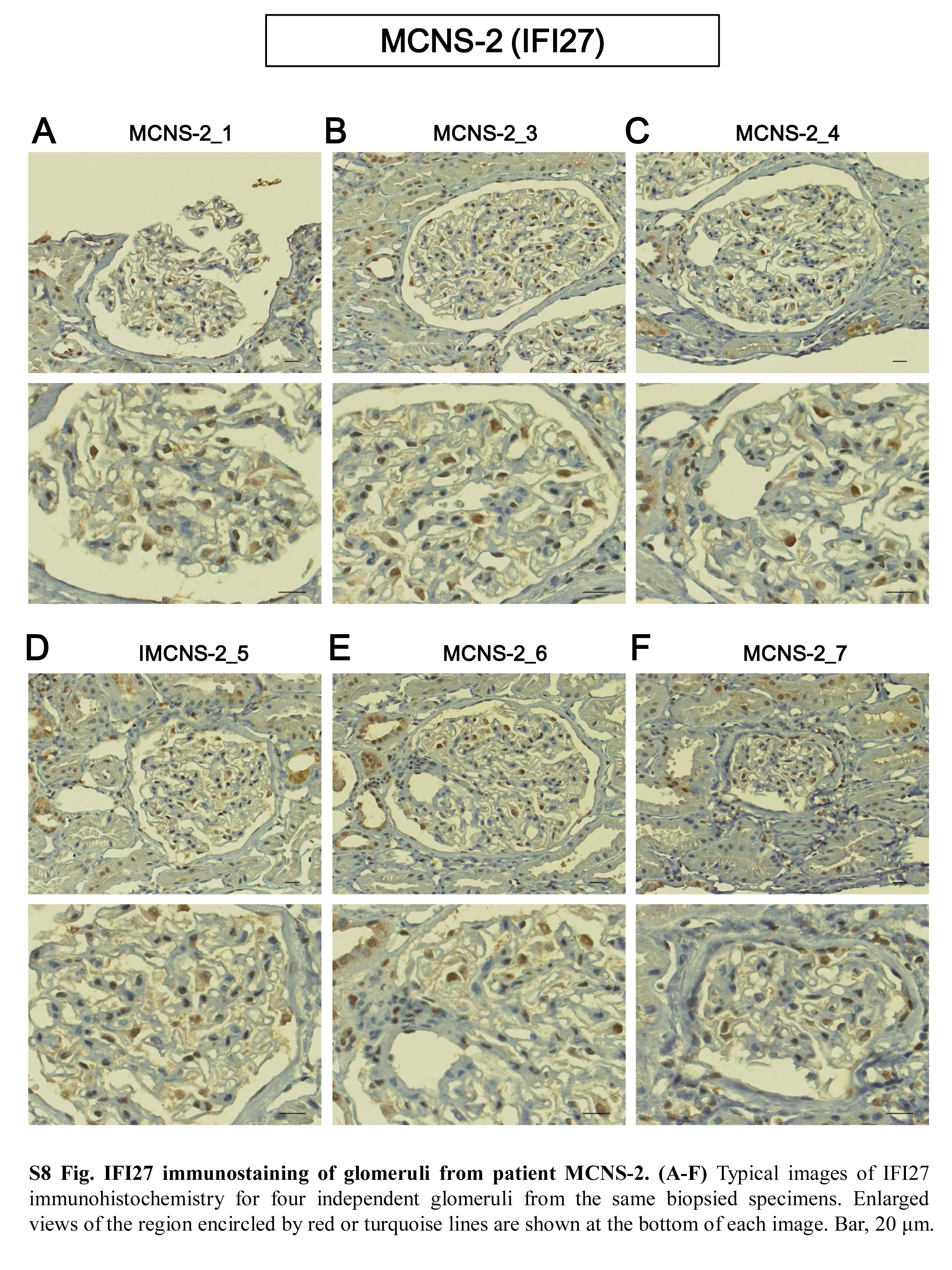

Supplement: S8 Fig — (A-F) Typical images of IFI27 immunohistochemistry for four independent glomeruli from the same biopsied specimens. Enlarged views of the region encircled by red or turquoise lines are shown at the bottom of each image. Bar, 20 μm. (TIF) [file pone.0153252.s008.tif]

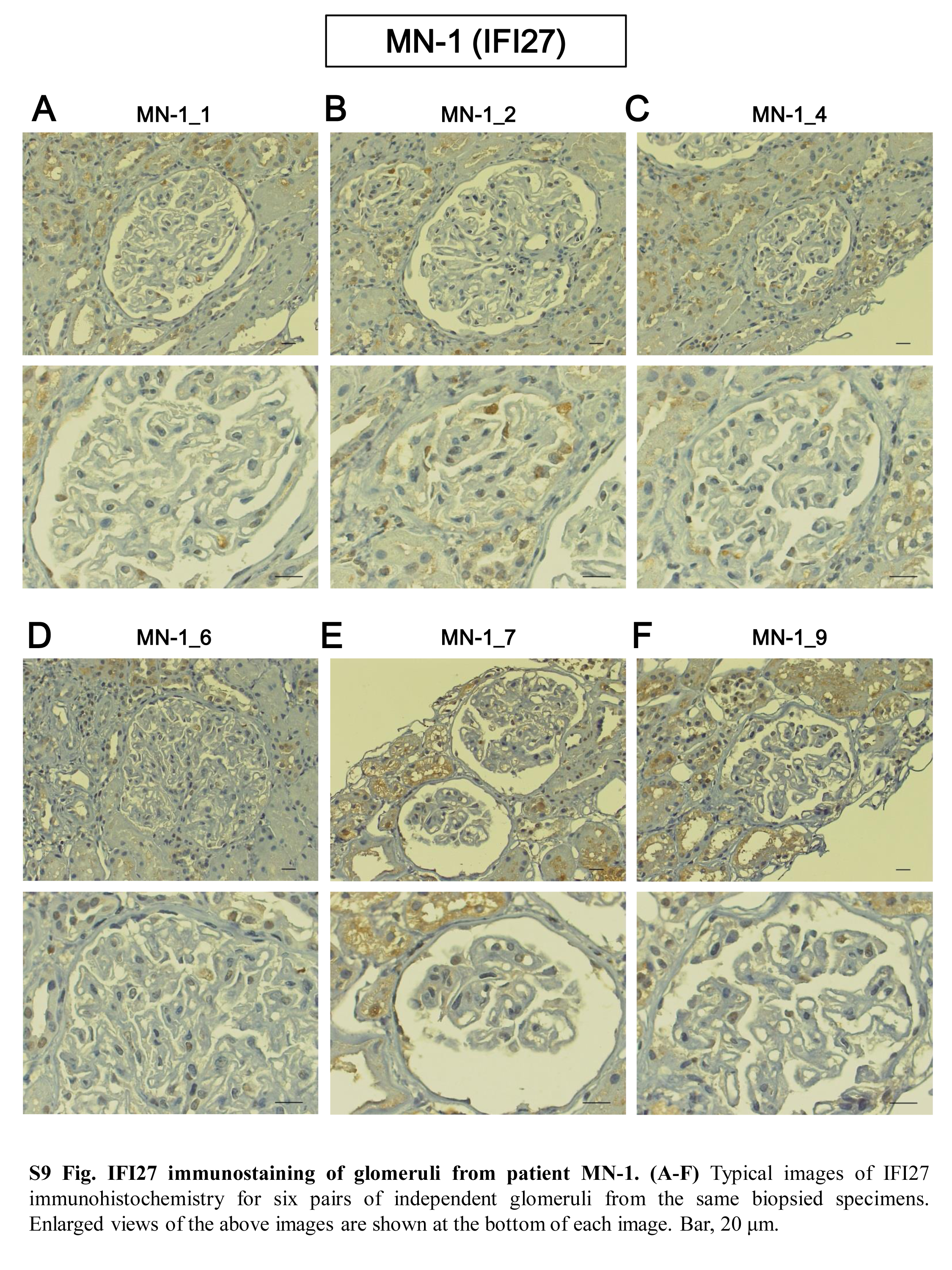

Supplement: S9 Fig — (A-F) Typical images of IFI27 immunohistochemistry for six pairs of independent glomeruli from the same biopsied specimens. Enlarged views of the above images are shown at the bottom of each image. Bar, 20 μm. (TIF) [file pone.0153252.s009.tif]

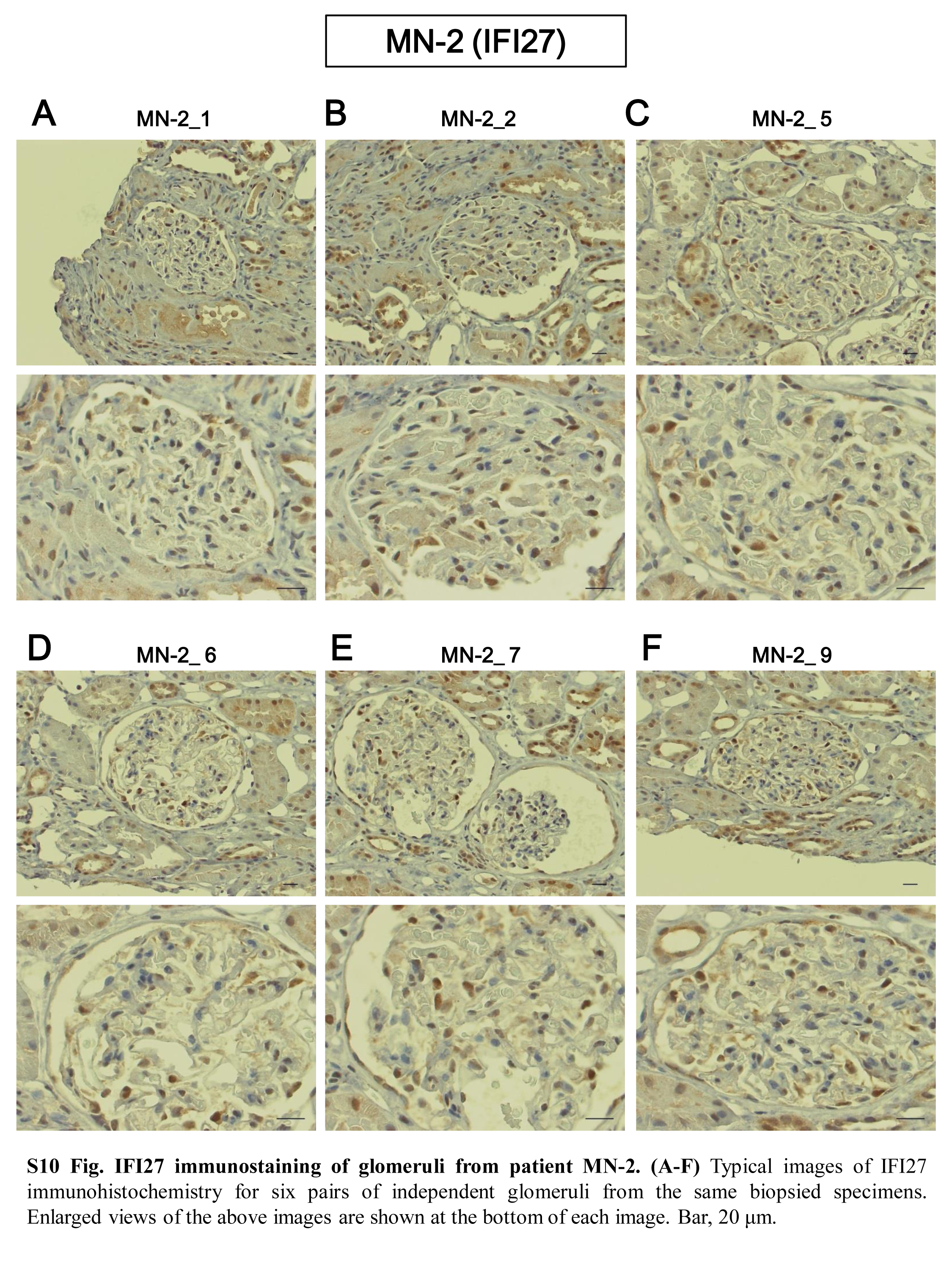

Supplement: S10 Fig — (A-F) Typical images of IFI27 immunohistochemistry for six pairs of independent glomeruli from the same biopsied specimens. Enlarged views of the above images are shown at the bottom of each image. Bar, 20 μm. (TIF) [file pone.0153252.s010.tif]

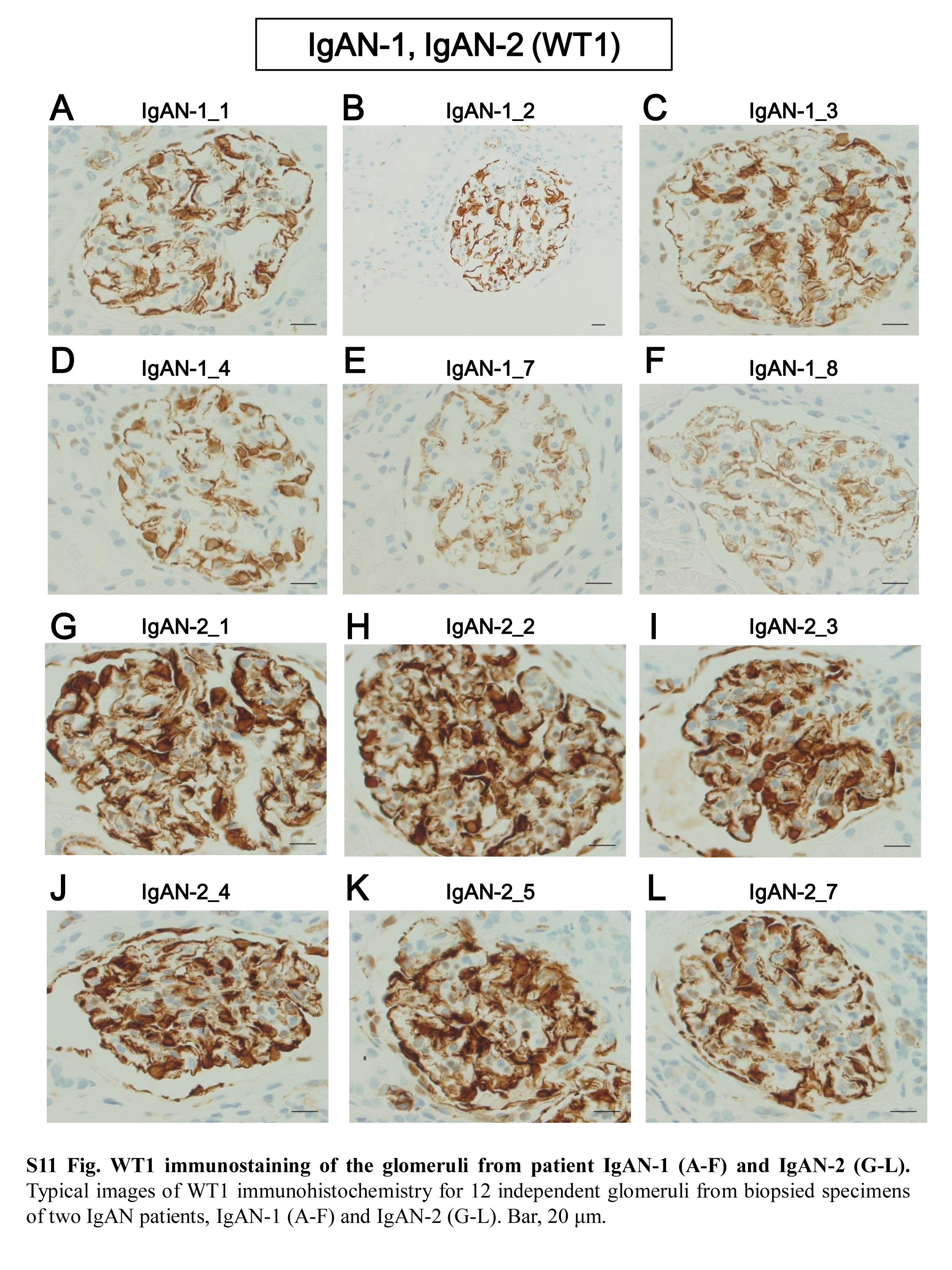

Supplement: S11 Fig — Typical images of WT1 immunohistochemistry for 12 independent glomeruli from biopsied specimens of two IgAN patients, IgAN-1 (A-F) and IgAN-2 (G-L). Bar, 20 μm. (TIF) [file pone.0153252.s011.tif]

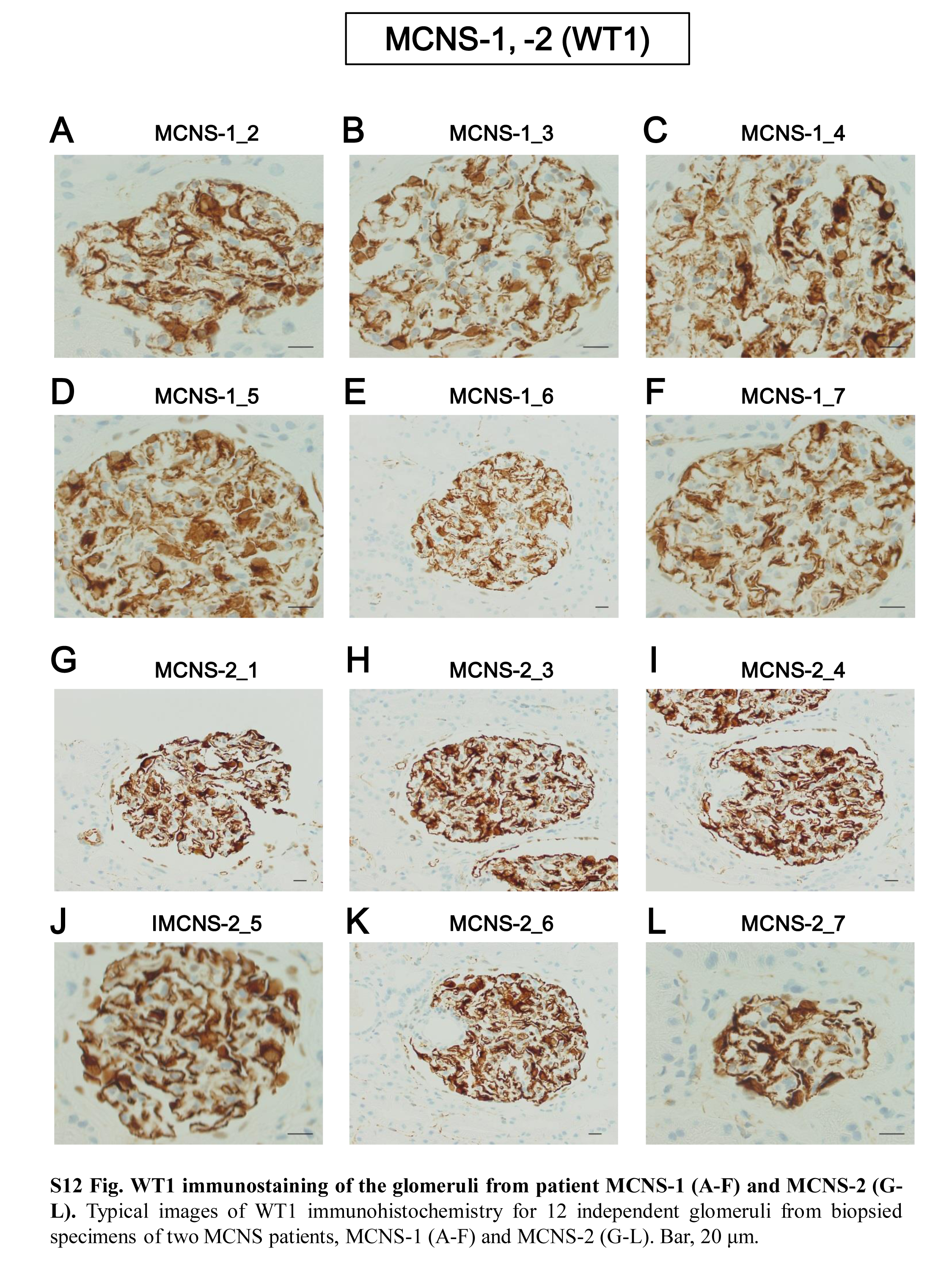

Supplement: S12 Fig — Typical images of WT1 immunohistochemistry for 12 independent glomeruli from biopsied specimens of two MCNS patients, MCNS-1 (A-F) and MCNS-2 (G-L). Bar, 20 μm. (TIF) [file pone.0153252.s012.tif]

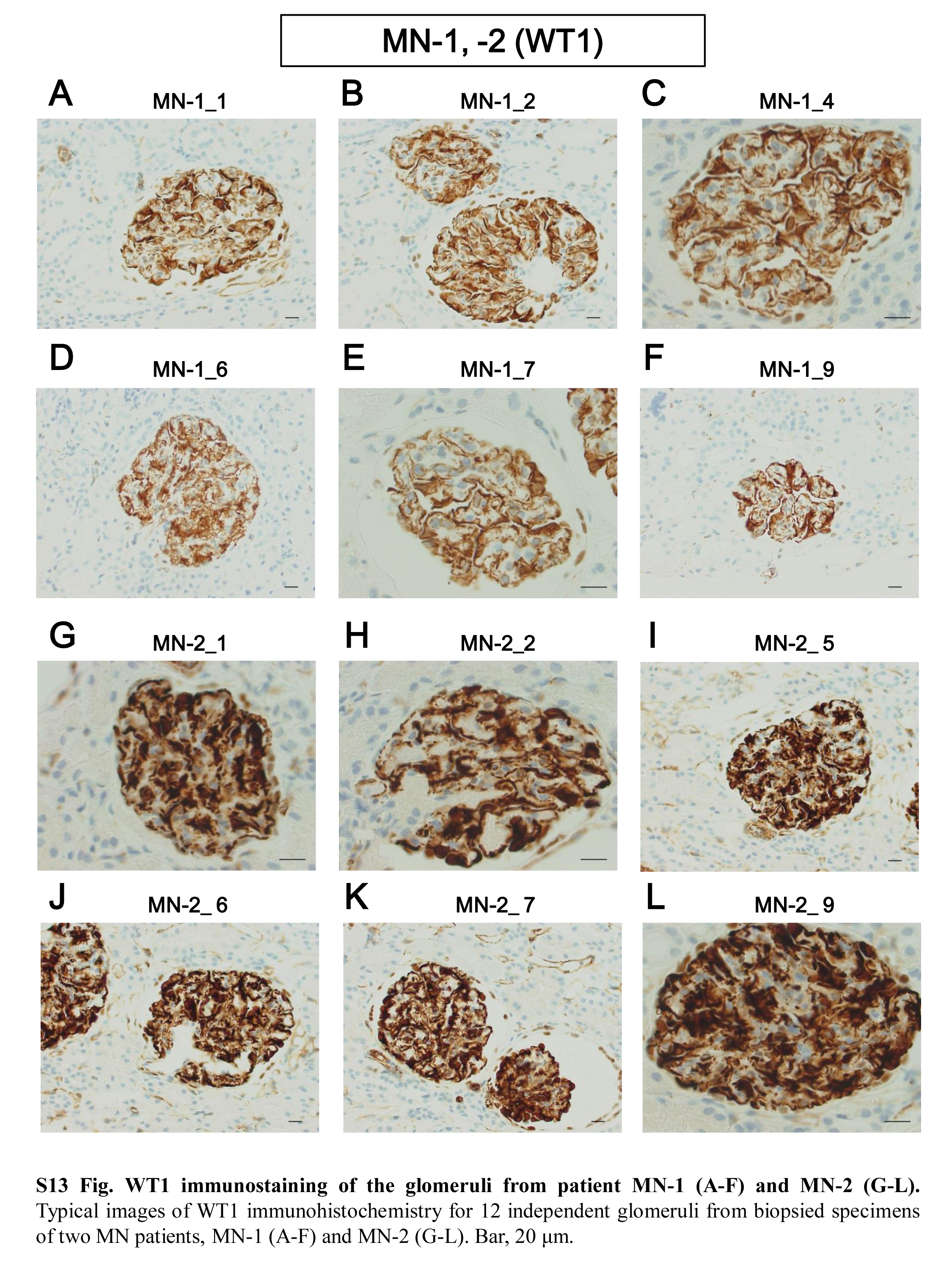

Supplement: S13 Fig — Typical images of WT1 immunohistochemistry for 12 independent glomeruli from biopsied specimens of two MN patients, MN-1 (A-F) and MN-2 (G-L). Bar, 20 μm. (TIF) [file pone.0153252.s013.tif]

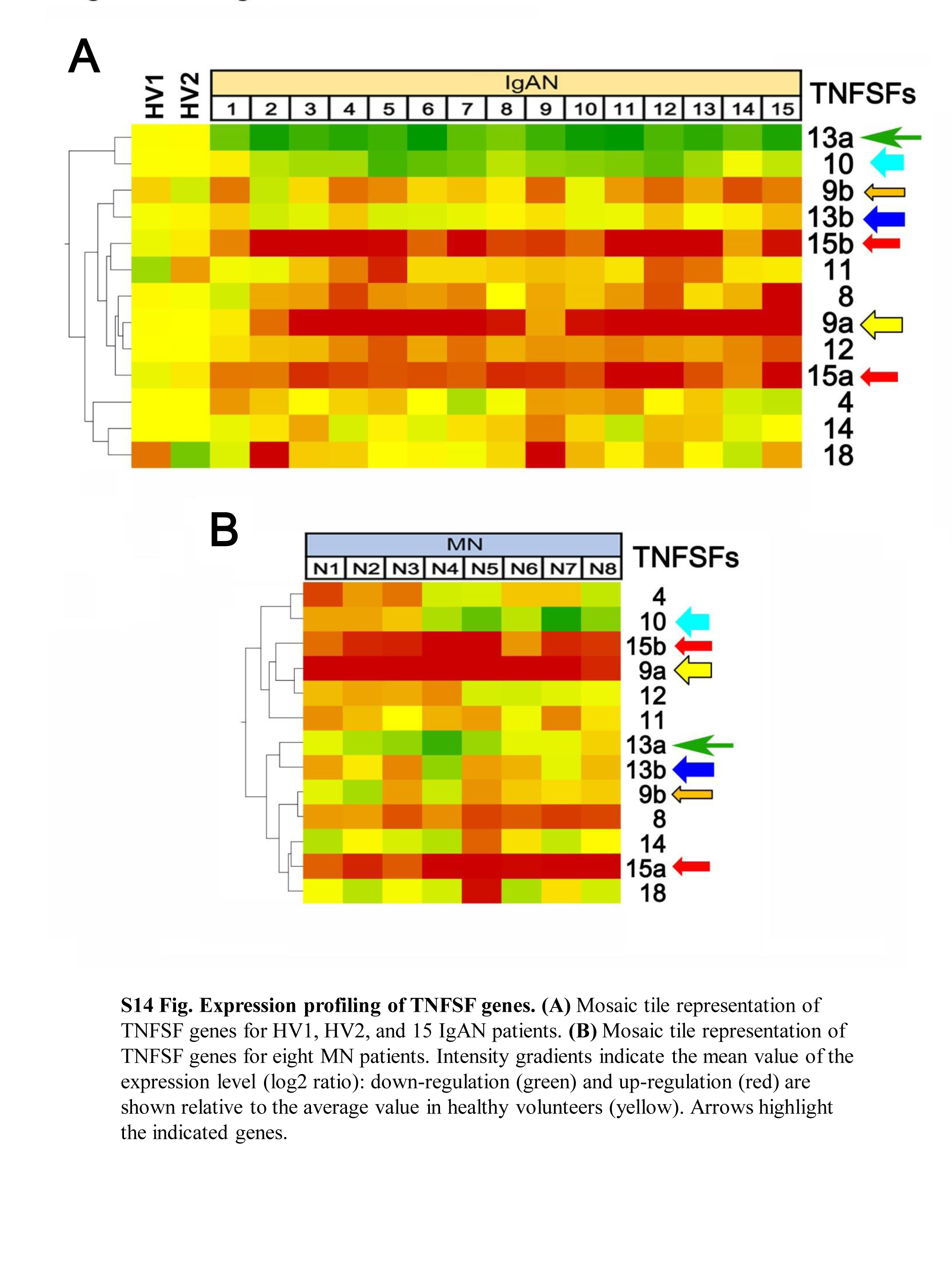

Supplement: S14 Fig — (A) Mosaic tile representation of TNFSF genes for HV1, HV2, and 15 IgAN patients. (B) Mosaic tile representation of TNFSF genes for eight MN patients. Intensity gradients indicate the mean value of the expression level (log2 ratio): down-regulation (green) and up-regulation (red) are shown relative to the average value in healthy volunteers (yellow). Arrows highlight the indicated genes. (TIF) [file pone.0153252.s014.tif]

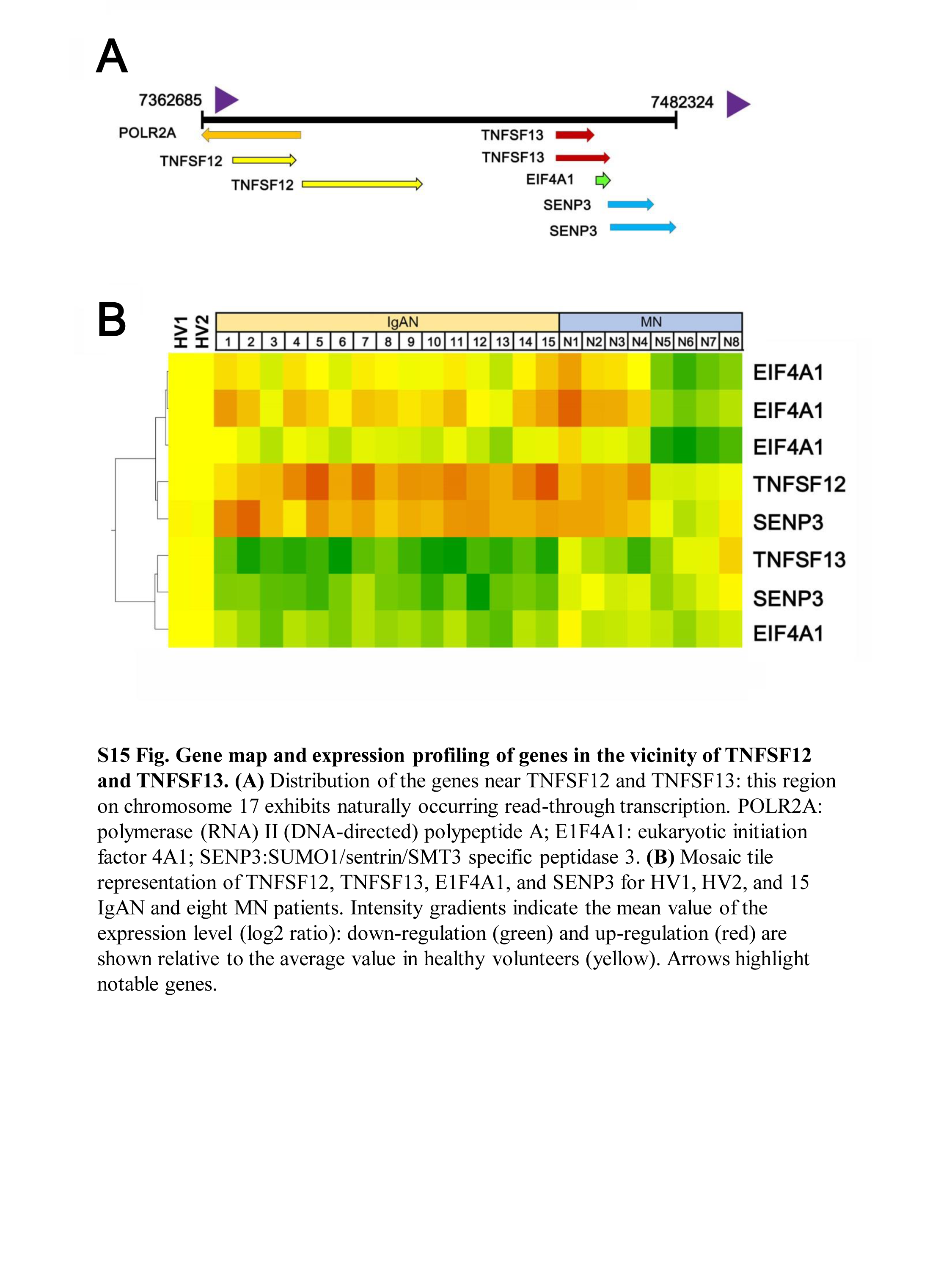

Supplement: S15 Fig — (A) Distribution of the genes near TNFSF12 and TNFSF13: this region on chromosome 17 exhibits naturally occurring read-through transcription. POLR2A: polymerase (RNA) II (DNA-directed) polypeptide A; E1F4A1: eukaryotic initiation factor 4A1; SENP3:SUMO1/sentrin/SMT3 specific peptidase 3. (B) Mosaic tile representation of TNFSF12, TNFSF13, E1F4A1, and SENP3 for HV1, HV2, and 15 IgAN and eight MN patients. Intensity gradients indicate the mean value of the expression level (log2 ratio): down-regulation (green) and up-regulation (red) are shown relative to the average value in healthy volunteers (yellow). Arrows highlight notable genes. (TIF) [file pone.0153252.s015.tif]

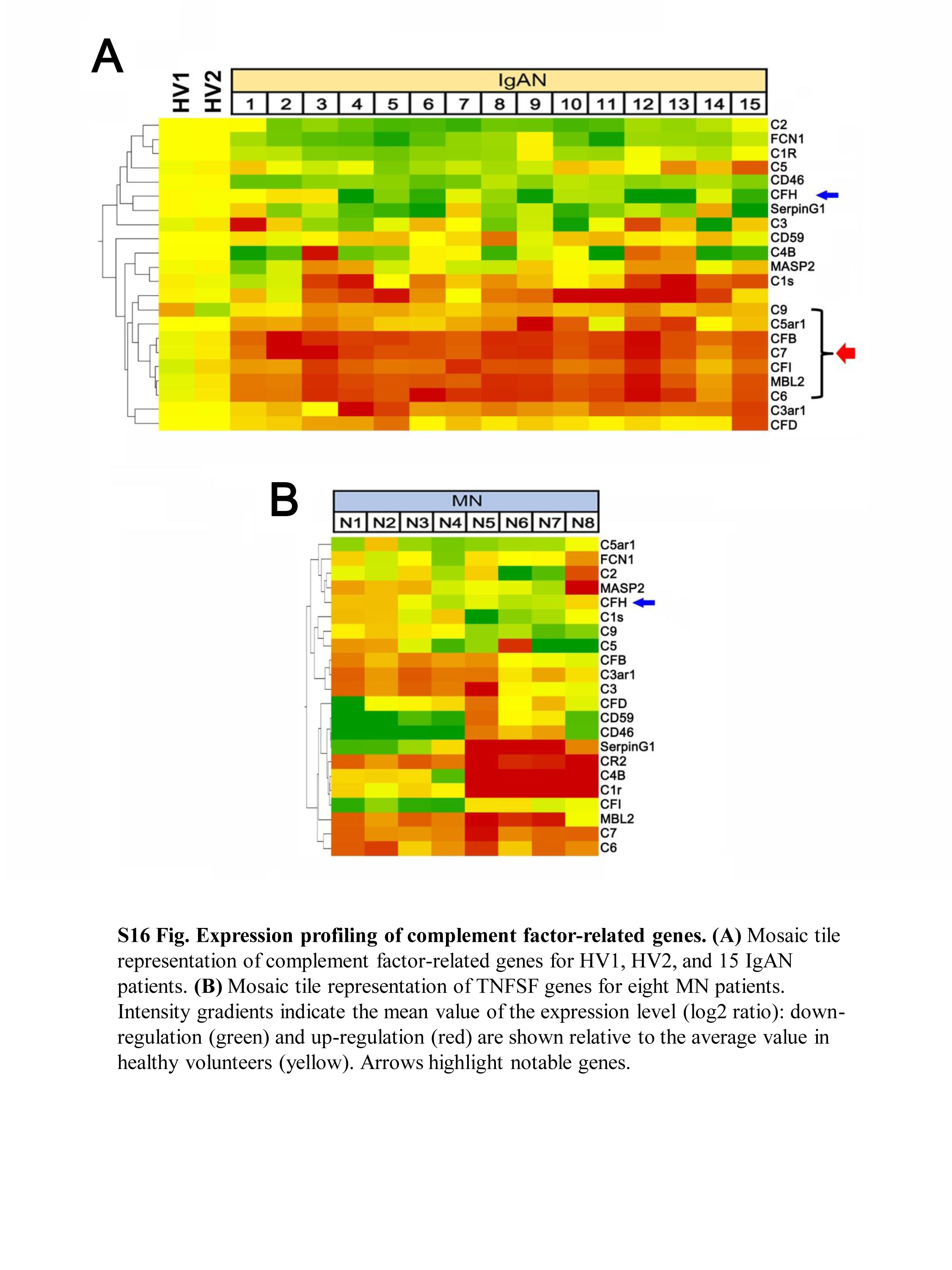

Supplement: S16 Fig — (A) Mosaic tile representation of complement factor-related genes for HV1, HV2, and 15 IgAN patients. (B) Mosaic tile representation of TNFSF genes for eight MN patients. Intensity gradients indicate the mean value of the expression level (log2 ratio): down-regulation (green) and up-regulation (red) are shown relative to the average value in healthy volunteers (yellow). Arrows highlight notable genes. (TIF) [file pone.0153252.s016.tif]

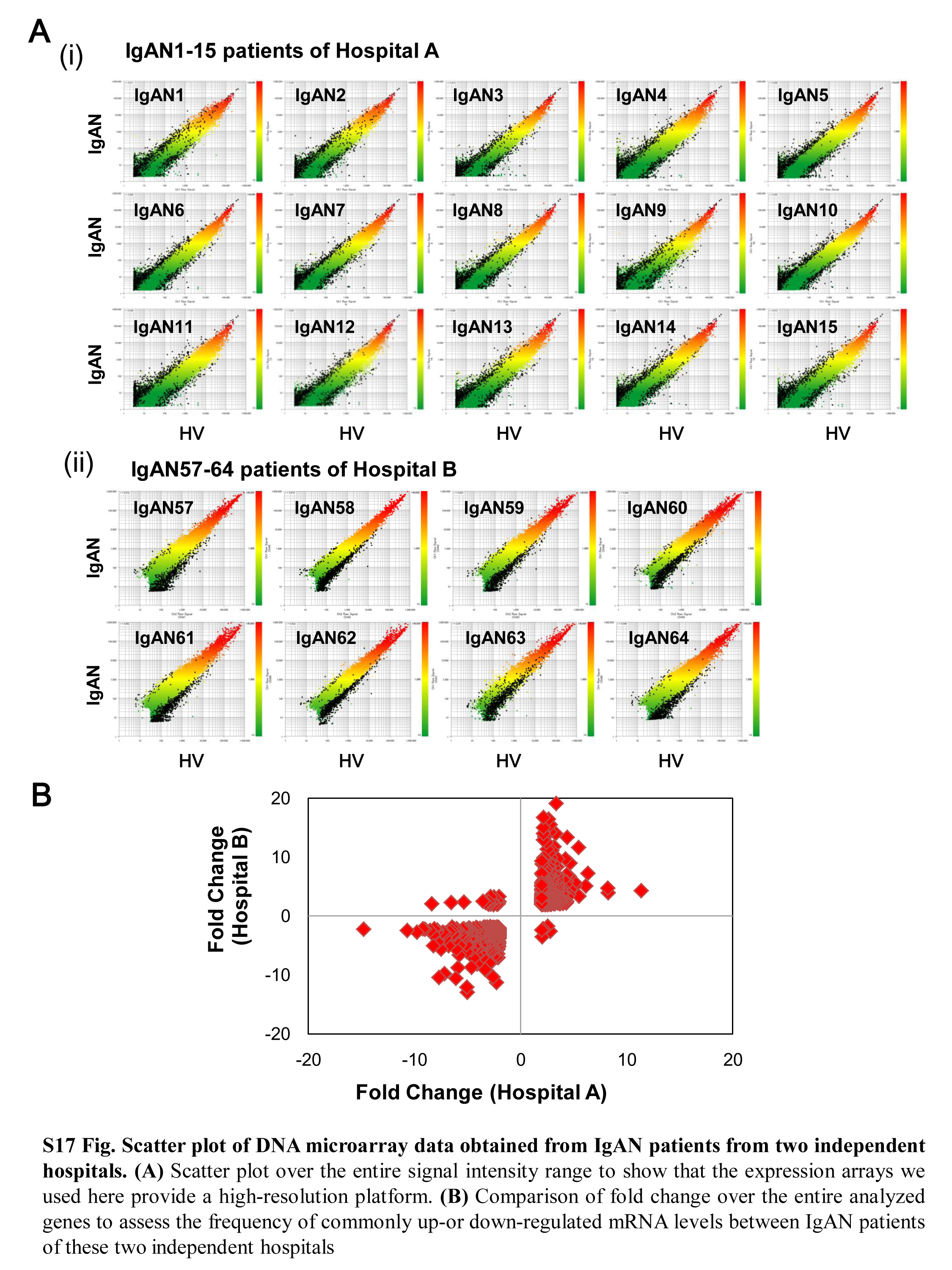

Supplement: S17 Fig — (A) Scatter plot over the entire signal intensity range to show that the expression arrays we used here provide a high-resolution platform. (B) Comparison of fold change over the entire analyzed genes to assess the frequency of commonly up-or down-regulated mRNA levels between IgAN patients of these two independent hospitals. (TIF) [file pone.0153252.s017.tif]

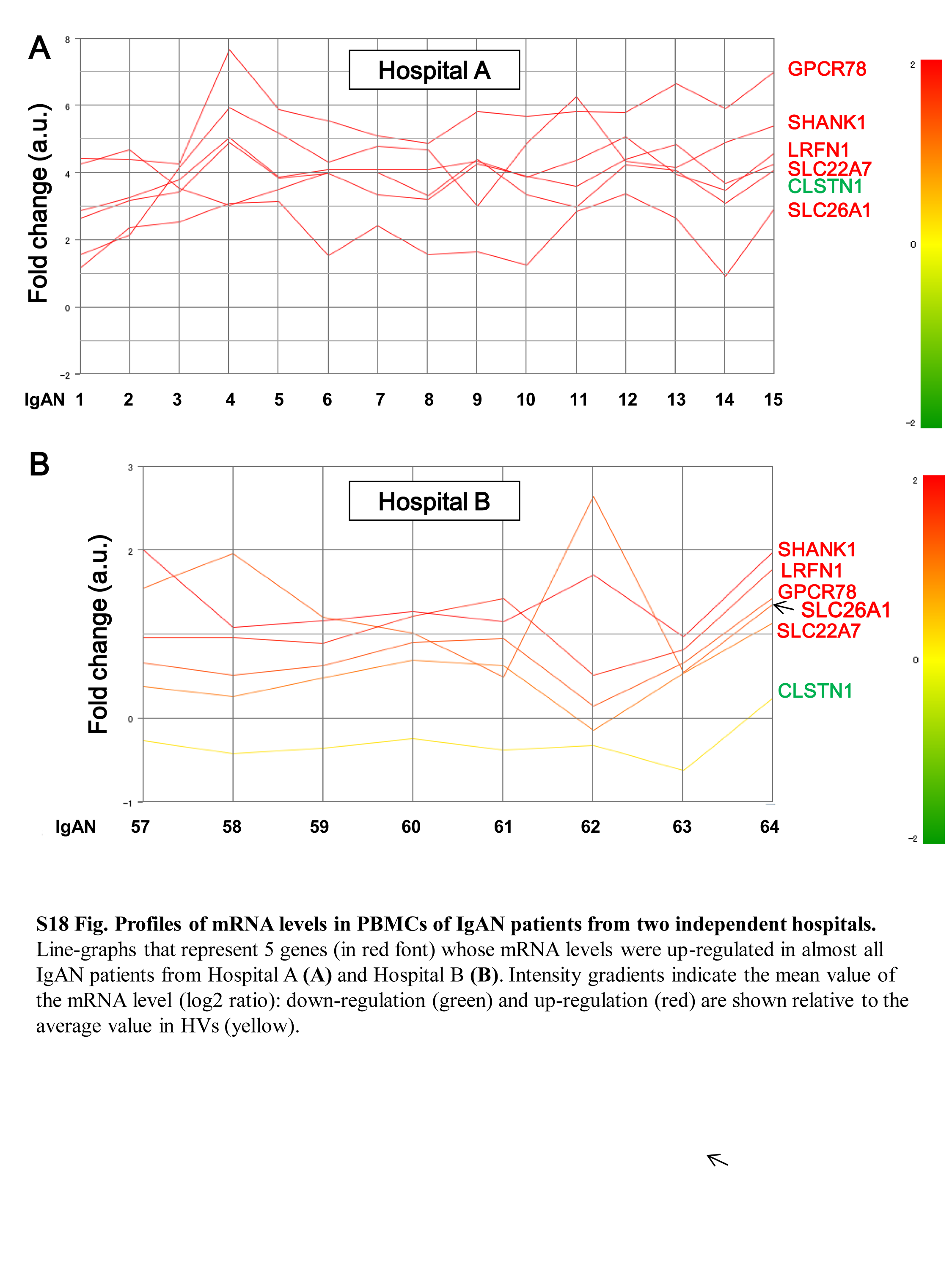

Supplement: S18 Fig — Line-graphs that represent 5 genes (in red font) whose mRNA levels were up-regulated in almost all IgAN patients from Osaka Univ. Hospital (A) and Kitano Hospital (B). Intensity gradients indicate the mean value of the mRNA level (log2 ratio): down-regulation (green) and up-regulation (red) are shown relative to the average value in HVs (yellow). (TIF) [file pone.0153252.s018.tif]
