# Supplementary material for: IFI27 Is a Useful Genetic Marker for Diagnosis of Immunoglobulin A Nephropathy and Membranous Nephropathy Using Peripheral Blood
Source: PLoS One. 2016 Apr 21;11(4):e0153252. doi: 10.1371/journal.pone.0153252 (PMC4839700; doi:10.1371/journal.pone.0153252)
Supplement: S1 Text — (DOCX) [file pone.0153252.s019.docx]

**Supplementary Results**

**Altered mRNA levels of *TNFSF* and complement factor genes in IgAN and MN patients**

A previous genome-wide association study (GWAS) in a South Chinese population identified the tumor necrosis factor ligand superfamily member 13 (*TNFSF13*) locus (Chromosome 17p13) as a susceptibility gene locus for serum IgA level in IgAN [34], [35]. Therefore, we examined *TNFSF13* mRNA levels in our DNA microarray data. We found that the level of *TNFSF13a* (transcript variant *a* of *TNFSF13* gene) was remarkably reduced in all IgAN patients relative to HVs (green arrow in S14A Fig). By contrast, this remarkable down-regulation was not observed in other members of TNFSF family, including *TNFSF13b* (blue arrow in S14A Fig), although *TNFSF10* was only weakly reduced (turquoise arrows in S10A Fig). Remarkably, *TNFSF15* and *TNFSF9a* (transcript variant *a* of *TNFSF9* gene) were conspicuously up-regulated in almost all IgAN patients (red, orange, and yellow arrows in S14A Fig). This reduced expression of *TNFSF13* was not due to deletion of this genomic region because mRNA levels of nearby genes (*TNFSF12* and transcript variants of *SENP3* and *EIF4a*) (S15A Fig) were elevated (S15B Fig).

Notably, reduced *TNFSF13* mRNA level was less conspicuous in MN patients (green-blue arrows in S14B Fig), and the *TNFSF10* mRNA level was weakly up-regulated in some MN patients (turquoise arrows in S14B Fig). By contrast, up-regulation of *TNFSF9b* mRNA level was not observed in some MN patients (orange arrows in S10A and S10B Figs). Taken together, these data suggest that *TNFSF13* and *TNFSF10* could serve as markers to distinguish IgAN from MN and HVs, whereas *TNFSF15* and *TNFSF9a* could serve as markers to distinguish IgAN and MN from HVs.

Because the GWAS also suggested complement factor h (CFH) as a susceptibility gene locus for serum IgA level in IgAN, we also examined the mRNA levels of complement factor genes. We found that CFH is reduced in some IgAN and MN patients relative to HVs (blue arrows in S16 Fig). By contrast, mRNA levels of other complement factors (collectively shown by the red arrow in S16A Fig) are up-regulated in almost all IgAN patients; this up-regulation was not observed in MN patients (S16B Fig). Thus, these complement factor genes may also serve as markers to distinguish IgAN from MN.

**Search for commonly up-regulated genes in PBMCs of IgAN patients from two independent hospitals**

To further search for the IgAN specific genes, we performed similar DNA microarray analysis using the RNA samples from eight IgAN patients (IgAN57-64; see Fig 4) of another hospital (Kitano Hospital). We obtained the relative signal intensity for each IgAN patient compared with those of HVs. When the data are plotted over the entire signal intensity range (scatter plot), we could demonstrate that these expression arrays provide a high-resolution platform (S17A Fig). Notably, mRNA levels of 30-41% genes are commonly up-or down-regulated between IgAN patients of these independent hospitals (S17B Fig).

Line graphs are shown for five genes (red font) whose mRNA levels are up-regulated in almost all IgAN patients from both hospitals (S18 Fig). They are G protein-coupled receptor 78 (GPCR78), SH3 and multiple ankyrin repeat domains protein 1 (SHANK1), leucine rich repeat and fibronectin type III domain containing protein 1 (LRFN1), solute carrier family 26A1 (SLC26A1) and solute carrier family 22A7 (SLC22A7). Calsyntenin-1 (CLSTN1) was up-regulated only in the patients from Osaka Uni. Hospital (S18A Fig). Unfortunately, we failed to match DNA data with those of qRT-PCR for these genes (data not shown). Thus, we conducted no further study on these genes.

**Supplementary References**

34. Kiryluk K, Novak J, Gharavi AG (2013) Pathogenesis of immunoglobulin A nephropathy: recent insight from genetic studies. Annu Rev Med 64: 339-356.

35. Yang C, Jie W, Yanlong Y, Xuefeng G, Aihua T, Yong G et al., (2012). Genome-wide association study identifies TNFSF13 as a susceptibility gene for IgA in a South Chinese population in smokers. Immunogenetics 64: 747-753.

**Figure Legends for Supporting Information**

**S1 Fig. Gender and age distributions of IgAN and MN patients.**

RNA purified from PBMCs of these patients was subjected to DNA microarray analysis. Females and males are indicated by circles and triangles, respectively.

**S2 Fig. Comparison of DNA microarray and qRT-PCR data.**

Relative mRNA levels, determined by DNA microarray **(A, C, E, G)** or qRT-PCR **(B, D, F, H)**, are shown as box graphs for *CLSTN* **(A, B)**, *COTL1* **(C, D)**, *KRTAP8-1* **(E, F)**, and *KRTAP5-8* **(G, H)** using RNA samples from individual IgAN and MN patients. Vertical axis indicates the mRNA level (arbitrary units: a.u.) relative to the value in HVs, which was fixed at 1.0 a.u.

**S3 Fig. Expression profiles of genes in PBMCs of 15 IgAN and eight MN patients. (A)** List of the top 31 genes up-regulated in most IgAN patients (fold-change >3.0), but not in MN patients (fold-change <1.1), shown in decreasing order of fold-change values for IgAN. *KRTAP5-8* (red font) was subjected to qRT-PCR analysis (see S2 Fig). **(B)** List of the bottom 22 genes down-regulated in most IgAN patients (fold-change <1.1), but not in MN patients (fold-change >3.0), shown in increasing order of fold-change values for IgAN. “Unknown” indicates uncharacterized genes. Mosaic tile representation for each gene is also shown, with intensity gradients indicating the mean value of the expression level (log_2_ ratio): down-regulation (green) and up-regulation (red) are expressed relative to the average value in healthy volunteers (yellow). **(C)** Bar represents the standard intensity gradient.

**S4 Fig. List of patients subjected to qRT-PCR analysis.**

**(A–C)** Patients correspond to those in Fig 4. **(D)** Patients correspond to those in Fig 6 and S5-13 Figs. Sample code number, abbreviated symbol, full description of disease names, fold-change values, age, and gender of each patient are shown.

**S5 Fig. IFI27 immunostaining of the glomeruli from patient IgAN-1.**

**(A-F)** Typical images of IFI27 immunohistochemistry for six pairs of independent glomeruli from the same biopsied specimens. Enlarged views of the above images are shown at the bottom of each image. Bar, 20 μm.

**S6 Fig. IFI27 immunostaining of glomeruli from patient IgAN-2.**

**(A-F)** Typical images of IFI27 immunohistochemistry for six pairs of independent glomeruli from the same biopsied specimens. Enlarged views of the above images are shown at the bottom of each image. Bar, 20 μm.

**S7 Fig. IFI27–immunostaining of the glomeruli from patient MCNS-1.**

**(A-F)** Typical images of IFI27 immunohistochemistry for six pairs of independent glomeruli from the same biopsied specimens. Enlarged views of the above images are shown at the bottom of each image. Bar, 20 μm.

**S8 Fig. IFI27 immunostaining of glomeruli from patient MCNS-2.**

**(A-F)** Typical images of IFI27 immunohistochemistry for four independent glomeruli from the same biopsied specimens. Enlarged views of the region encircled by red or turquoise lines are shown at the bottom of each image. Bar, 20 μm.

**S9 Fig. IFI27 immunostaining of glomeruli from patient MN-1.**

**(A-F)** Typical images of IFI27 immunohistochemistry for six pairs of independent glomeruli from the same biopsied specimens. Enlarged views of the above images are shown at the bottom of each image. Bar, 20 μm.

**S10 Fig. IFI27 immunostaining of glomeruli from patient MN-2.**

**(A-F)** Typical images of IFI27 immunohistochemistry for six pairs of independent glomeruli from the same biopsied specimens. Enlarged views of the above images are shown at the bottom of each image. Bar, 20 μm.

**S11 Fig. WT1 immunostaining of the glomeruli from patient IgAN-1 (A-F) and IgAN-2 (G-L).**

Typical images of WT1 immunohistochemistry for 12 independent glomeruli from biopsied specimens of two IgAN patients, IgAN-1 (A-F) and IgAN-2 (G-L). Bar, 20 μm.

**S12 Fig. WT1 immunostaining of the glomeruli from patient MCNS-1 (A-F) and MCNS-2 (G-L).**

Typical images of WT1 immunohistochemistry for 12 independent glomeruli from biopsied specimens of two MCNS patients, MCNS-1 (A-F) and MCNS-2 (G-L). Bar, 20 μm.

**S13 Fig. WT1 immunostaining of the glomeruli from patient MN-1 (A-F) and MN-2 (G-L).**

Typical images of WT1 immunohistochemistry for 12 independent glomeruli from biopsied specimens of two MN patients, MN-1 (A-F) and MN-2 (G-L). Bar, 20 μm.

**S14 Fig. Expression profiling of TNFSF genes.**

**(A)** Mosaic tile representation of TNFSF genes for HV1, HV2, and 15 IgAN patients. **(B)** Mosaic tile representation of TNFSF genes for eight MN patients. Intensity gradients indicate the mean value of the expression level (log2 ratio): down-regulation (green) and up-regulation (red) are shown relative to the average value in healthy volunteers (yellow). Arrows highlight the indicated genes.

**S15 Fig. Gene map and expression profiling of genes in the vicinity of TNFSF12 and TNFSF13. (A)** Distribution of the genes near TNFSF12 and TNFSF13: this region on chromosome 17 exhibits naturally occurring read-through transcription. POLR2A: polymerase (RNA) II (DNA-directed) polypeptide A; E1F4A1: eukaryotic initiation factor 4A1; SENP3:SUMO1/sentrin/SMT3 specific peptidase 3. **(B)** Mosaic tile representation of TNFSF12, TNFSF13, E1F4A1, and SENP3 for HV1, HV2, and 15 IgAN and eight MN patients. Intensity gradients indicate the mean value of the expression level (log2 ratio): down-regulation (green) and up-regulation (red) are shown relative to the average value in healthy volunteers (yellow). Arrows highlight notable genes.

**S16 Fig. Expression profiling of complement factor-related genes.**

**(A)** Mosaic tile representation of complement factor-related genes for HV1, HV2, and 15 IgAN patients. **(B)** Mosaic tile representation of TNFSF genes for eight MN patients. Intensity gradients indicate the mean value of the expression level (log2 ratio): down-regulation (green) and up-regulation (red) are shown relative to the average value in healthy volunteers (yellow). Arrows highlight notable genes.

**S17 Fig. Scatter plot of DNA microarray data obtained from IgAN patients from two independent hospitals.**

**(A)** Scatter plot over the entire signal intensity range to show that the expression arrays we used here provide a high-resolution platform. **(B)** Comparison of fold change over the entire analyzed genes to assess the frequency of commonly up-or down-regulated mRNA levels between IgAN patients of these two independent hospitals

**S18 Fig. Profiles of mRNA levels in PBMCs of IgAN patients from two independent hospitals.**

Line-graphs that represent 5 genes (in red font) whose mRNA levels were up-regulated in almost all IgAN patients from Osaka Univ. Hospital **(A)** and Kitano Hospital **(B)**. Intensity gradients indicate the mean value of the mRNA level (log2 ratio): down-regulation (green) and up-regulation (red) are shown relative to the average value in HVs (yellow).
